# Nucleosome Assembly Protein 1 in Dinoflagellate Cells Without Architectural Nucleosomes

**DOI:** 10.3390/ijms27177931

**Published:** 2026-09-05

**Authors:** Kosmo Ting Hin Yan, Alvin Chun Man Kwok, Shaoping Wen, Fang Zhang, Joseph Tin Yum Wong

**Affiliations:** Division of Life Science, The Hong Kong University of Science and Technology, Clearwater Bay, Kowloon, Hong Kong, China; kosmo@connect.ust.hk (K.T.H.Y.); alvink@ust.hk (A.C.M.K.); wensprussell@gmail.com (S.W.); fzhangah@connect.ust.hk (F.Z.)

**Keywords:** nucleosome assembly factor, core histones, histone-like protein, histone H1 superfamily, dinoflagellates, cell cycle, chromosome ionotropic environment

## Abstract

Nucleosome assembly protein 1 (NAP1) is highly conserved across eukaryotes, yet its biochemical function, particularly its proposed role as a histone chaperone, remains unresolved. Dinoflagellates, including *Karenia brevis* (*Kb*) and *Crypthecodinium cohnii* (*Cc*), lack architectural nucleosomes and express core histones at unusually low levels, yet retain abundant *NAP1* transcripts encoding two to three distinct homologs. Confocal immunolocalization of *K. brevis* showed strong nuclear signals for both homologs, between chromosomes and at the nucleolus, with higher nuclear-to-cytoplasmic ratios in G_2_ than in G_1_ (the two gap phases of the cell cycle); labeling at chromosome-territory margins and at the nuclear cortex is consistent with an association with the telomeric nucleosomes anchored to the nuclear envelope. Recombinant KbNAP1Bp reproduced the canonical yeast NAP1 fold, recovered H2A-immunoreactive material in immunoprecipitation, and preferentially retarded larger DNA fragments in gel mobility assays, whereas KbNAP1Ap did none of these under the conditions tested. In *C. cohnii*, the NAP1 protein peaked at S–G_2_, and exposure to a *CcNAP1.1*-antisense oligodeoxynucleotide (ODN) was associated with an S–G_2_/M delay. These findings uncouple NAP1 abundance from the availability of a canonical nucleosomal substrate and suggest evolutionary repurposing toward non-nucleosomal roles in chromosome-territory organization. They help define the minimal functional core of this conserved chaperone family and caution against treating NAP1 abundance as a proxy for nucleosome assembly activity.

## 1. Introduction

Dinoflagellate nuclei lack canonical architectural nucleosomes, compacting their genomes instead through extensive DNA plectonemes that generate the cholesteric liquid-crystalline organization of the permanently condensed dinokaryon [1,2]. Despite this secondary loss of nucleosomal architecture and the correspondingly low transcript levels of canonical replication-dependent histones [3,4], nucleosome assembly protein 1 (NAP1) homologs are expressed at relatively high levels in dinoflagellate transcriptomes (Section 2.1). NAP1 proteins are widely regarded as multifunctional histone chaperones, with reported roles in H2A variant exchange [5], linker histone deposition [6], nucleosome sliding [7], histone shuttling [8], transcription regulation [9,10], promotion of octamer assembly in vitro [11,12], prevention of non-specific histone–DNA interactions [13] and, more recently, chromatin phase transitions [14]. Family members are not invariably nuclear, since the *Chlamydomonas reinhardtii* homolog cNAPL carries a 46-residue chloroplast-targeting leader peptide [15], so NAP1 activity cannot be assumed a priori to be restricted to nucleosome assembly. Because nucleosome architecture is a central determinant of chromatin accessibility and gene regulation, and its dysregulation contributes to disease states, including cancer [16], how NAP1 protein operates is of broad interest. The retention of abundant NAP1 transcripts in an organism whose nucleosomal substrate is severely diminished, therefore, raises a fundamental question about the protein’s non-nucleosomal functions.

In dinoflagellates, the most abundant chromosomal proteins are not histones but a family of small basic proteins, the dinoflagellate histone-like proteins (dHLPs), related to bacterial HU-type DNA-binding proteins [17]. Their localization to peripheral chromatin loops (PCLs) [18] and their concentration-dependent capacity to reconfigure DNA suggest a central role in transcriptional organization [19,20]. Given the virtual absence of core histones, an obvious possibility is that NAP1 acts as a surrogate chaperone for these proteins. Their basic residues, however, are concentrated in a C-terminal helix, the region that carries DNA-binding activity in HCc3 [17,19,20], rather than in a centrally located histone-fold surface of the kind presented by the H2A–H2B heterodimer and are preferentially engaged by the NAP1 earmuff domain [21]. The canonical recognition mode is therefore unlikely to apply directly. The charge properties of the two partners also make this interaction intrinsically difficult to assay. Dinoflagellate NAP1p is strongly acidic (KbNAP1B, calculated pI ≈ 4.83, net charge ≈ −25.7 at pH 7) and the dHLPs strongly basic (HCc3, calculated pI ≈ 10.18, net charge ≈ +17.1 at pH 7; Section 4.2.2), so association in vitro is expected on electrostatic grounds irrespective of biological specificity.

NAP1 is also involved in nucleocytoplasmic trafficking. Nuclear transport relies mainly on classical importin/exportin–RanGTP systems [22,23,24,25,26], although non-classical pathways exist [27]. NAP1 participates both as cargo and as a co-factor for histone import [25], and its distribution between compartments is an active, cell-cycle-regulated process [28] governed by the balance of the NLS and NES motifs, with NES-mediated export typically dominating, and by CK2-dependent phosphorylation of NLS-masking domains [29]. NAP1, accordingly, performs diverse cell-cycle-dependent roles spanning chromatin assembly, transcription and nucleosomal dynamics [30,31]. Its compartmental distribution is therefore an actively maintained variable rather than a fixed property and cannot be taken to report a histone-chaperoning function.

Dinoflagellate nuclei carry up to ~200 pg DNA at a DNA:protein ratio of up to 10:1, rather than the typical eukaryotic ~1:1 [32,33], and their chromosomes require Ca^2+^ and Mg^2+^ for structural stabilization [34]. Secondary ion mass spectrometry of *Prorocentrum micans* nuclei gives ~0.6 divalent cations per base pair at a Mg^2+^:Ca^2+^ ratio of ~3:1 [35], roughly an order of magnitude above human nuclei (~one Ca^2+^ per 12.5–20 bases, one Mg^2+^ per 20–30 bases) [36], and close to the ~0.63 divalent ions per base pair at which protein-free DNA is charge-neutralized and maximally condensed in a two-dimensional model system [37]. This near-coincidence underpins the proposal that dinoflagellate DNA is condensed substantially by direct cation action within a liquid-crystalline framework [35], although the subsequent identification of divergent histones, dHLPs and dinoflagellate viral nucleoproteins (DVNPs) shows a purely electrostatic model to be an oversimplification [4,38]. In such a nucleus any abundant mobile polyanion is, in principle, a competitor for the divalent cations and basic proteins that stabilize the condensed state. We therefore raise, as a hypothesis to be tested rather than a conclusion, that dNAP1 contributes to chromatin organization through low-specificity polyanionic shielding in addition to, or instead of, canonical chaperoning.

Persistence of the nuclear envelope through dinomitosis adds a second, non-electrostatic consideration. Because nuclear and cytoplasmic pools are never rendered continuous by envelope breakdown, the partitioning of an abundant protein must be maintained by nucleocytoplasmic transport at every cell-cycle stage, as it is for ScNap1, which shuttles between compartments [28], and whose CK2-site mutants show prolonged S phase and altered nuclear import [39]. The central aim of this study was therefore to determine whether dNAP1 is a functional, cell-cycle-coupled chromatin protein in a nucleus that has largely lost its canonical substrate and to establish when and where it acts. Exploiting the highly synchronizable cultures obtainable from *Crypthecodinium cohnii* and the large, morphologically tractable nucleus of *Karenia brevis*, we asked (i) whether dNAP1 forms cell-cycle-dependent associations with DNA, histones and chromosome territories; (ii) whether its nucleocytoplasmic distribution is cell-cycle regulated; and (iii) whether antisense interference directed at a single homolog, CcNAP1.1, is sufficient to perturb S/G_2_ progression.

## 2. Results

### 2.1. Dinoflagellate NAP1 Homologs (dNAP1s): Sequence Diversity and Predicted Nuclear Trafficking Signals

**Figure 1:**
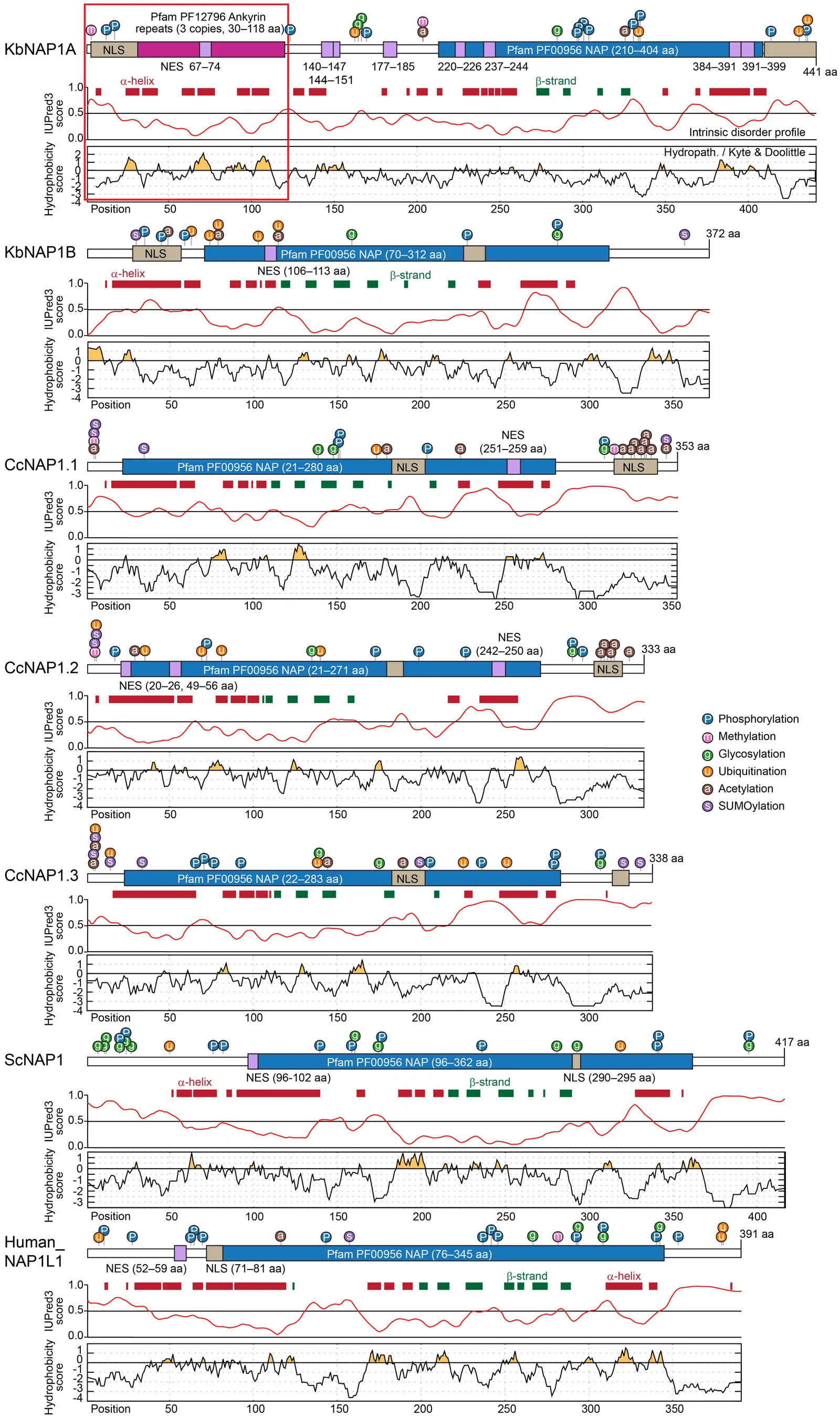
In silico comparisons among dinoflagellate and selected eukaryotic NAP1 homologs. Schematic of predicted domain organization, post-translational modification (PTM) sites, intrinsic disorder and Kyte–Doolittle hydrophobicity for NAP1 homologs from *Karenia brevis* (KbNAP1A, 441 aa; KbNAP1B, 372 aa), *Crypthecodinium cohnii* (CcNAP1.1, 353 aa; CcNAP1.2, 333 aa; CcNAP1.3, 338 aa), *Saccharomyces cerevisiae* (ScNAP1, 417 aa) and *Homo sapiens* (Human_NAP1L1, 391 aa). Predicted secondary structure is shown as solid boxes: α-helices (red), β-strands (green) and the conserved NAP1 domain (Pfam PF00956; blue). The N-terminal ankyrin-repeat cluster unique to KbNAP1A among the sequences shown (Pfam PF12796; three copies, residues 30–118) is outlined by a red dashed box. Predicted PTMs: P, phosphorylation; Ub, ubiquitination; Ac, acetylation; Me, methylation; Sumo, SUMOylation (MusiteDeep, score ≥ 0.5; sequence contexts in Table S4). Predicted positions of nuclear localization signals (NLSs) and nuclear export signals (NES) are labelled above each schematic. AIUPred disorder scores are shown as a red line (right y-axis); residues scoring > 0.5 are classified as intrinsically disordered and are concentrated toward the C-terminus in all homologs. Kyte–Doolittle hydrophobicity is shown as a black line (right y-axis), with values > 0 highlighted in orange. All homologs show low overall hydrophobicity, consistent with their predominantly acidic character (pI 4.4–4.8) and with their soluble rather than membrane-associated character. Total length (aa) is given at the right of each schematic. The complete alignment is provided in Figure S1.

Every dinoflagellate transcriptome examined (*n* = 10) yielded two to three sequences containing a recognizable NAP1 domain (44–58% pairwise similarity within the domain; Figure 1), despite the characteristically low core-histone transcript levels of this group [4]. In *C. cohnii*, the two dominant homologues *CcNAP1.2* and *CcNAP1.3* (FPKM 45 and 28 at G_2_/M) exceeded *CcNAP1.1* (FPKM 1.0) by 45- and 28-fold, respectively. Summed d*NAP1* transcript abundance reached 30–40% of that of the d*HLP*s, the most prevalent basic chromosomal proteins in dinoflagellates, and therefore exceeded core-histone transcript levels, which lie approximately an order of magnitude below the d*HLP*s [4]. The plurality of homologs seen here and across eukaryotic genomes is nonetheless counterintuitive if NAP1 functions solely as an H2A:H2B transport adaptor.

One naming convention is applied throughout, without exception. Isoform designations (*CcNAP1.1–1.3*, *KbNAP1A* and *KbNAP1B*) denote nucleotide sequences: the transcriptome-derived sequences described in this section and, in Section 2.5, the transcript targeted by the antisense ODN. Recombinant *K. brevis* proteins are designated KbNAP1Ap and KbNAP1Bp; the corresponding antibodies, anti-KbNAP1A and anti-KbNAP1B; and protein species detected in *C. cohnii* extracts are identified only by the antibody that detects them together with their apparent mass (for example, “anti-KbNAP1A-reactive ~69 kDa species”), because these antibodies cannot resolve the three CcNAP1 paralogs (Section 2.5). The designations CcNAP1.1p, CcNAP1.2p and CcNAP1.3p are not used.

The GenBank TSA database contained two *K. brevis* NAP1 homologs: KbNAP1A (441 aa, predicted 49.8 kDa, pI 4.7) and KbNAP1B (372 aa, predicted 43.2 kDa, pI 4.8), sharing only 34% amino-acid similarity. Both carried predicted NLS and NES sequences (Figure 1 and Figure S1) [28,40]. KbNAP1A contained N-terminal ankyrin repeats (residues 30–118), also present in *Karenia mikimotoi* (GJRC01030962.1), *Dinophysis acuminata* (GKBP01054707.1) and *Gambierdiscus pacificus* (GIJQ01024740.1), as well as in certain higher-plant NAP1-related proteins (*Brassica* spp.: A0A3P6C3Q2_BRACM, M4D1T9_BRARP and A0A078FN44_BRANA), in which this ankyrin-containing extension falls within the NAP1-related protein (NRP) domain implicated in nuclear localization and homo/heterodimerization [41]. KbNAP1A also carried a longer putative dimerization domain and a shorter C-terminal acidic domain (acidic domain 3) than the other homologs (Figure S1). All sequences shared the high acidic-residue content and pI of 4–5 characteristic of NAP1 proteins across taxa, which accounts for their anomalous SDS-PAGE migration independently of post-translational modification [42,43,44,45]. For direct comparison with a metazoan ortholog, human NAP1L1 is included in Figure 1 and Figure S1. Amino-acid identity between the dinoflagellate homologs and human NAP1L1 is low, ranging from 13.7% to 19.2% for KbNAP1A/B and 19.9% to 22.4% for CcNAP1.1–1.3. Sequence divergence is concentrated in the intrinsically disordered terminal regions and in the KbNAP1A-specific N-terminal ankyrin-repeat extension, which has no human counterpart.

To survey nuclear trafficking signals systematically, we screened 33 dinoflagellate NAP1 sequences against the Eukaryotic Linear Motif resource (ELM; http://elm.eu.org) [46] alongside 20 comparator sequences from human, fungi and plants (Figures S2 and S3; Section 4.2.1). Because no dinoflagellate motif instances are curated in ELM, all matches are pattern-based candidates rather than validated instances. At least one NLS class was matched in 30/33 dinoflagellate sequences (90.9%), dominated by the N-extended monopartite variant (TRG_NLS_MonoExtN_4; 29/33, 87.9%), which lies within the mid-to-C-terminal functional core, consistent with the structurally characterized β-hairpin NLS of yeast Nap1 [47]; a bipartite NLS (TRG_NLS_Bipartite_1) was matched in only 6/33 sequences (18.2%), against 3/4 human sequences (75.0%). At least one CRM1-dependent NES class was matched in 13/33 sequences (39.4%), and 10/33 (30.3%), including CcNAP1.1, CcNAP1.2 and KbNAP1A, carried both an NLS and an NES, a configuration compatible with nucleocytoplasmic shuttling; four sequences (12.1%) matched neither class, a category absent from the human, plant and fungal comparators. Per-class counts, per-sequence positions and group denominators are given in Figures S2 and S3; all values are descriptive frequencies within the sets analyzed and were not tested for statistical enrichment (Section 4.2.3).

Our two anti-NAP1 antibodies, raised against bacterially expressed KbNAP1A (residues 145–441, lacking the ankyrin repeat) and KbNAP1B and affinity-purified against their respective antigens (Section 4.4), showed no cross-reactivity (Figure S4A–C). The presence of multiple NES sequences in KbNAP1A and CcNAP1.2 may create preferred orientations in solution and thereby influence surface exposure on immunoblot membranes. Anti-KbNAP1A recognized both full-length KbNAP1Ap and the ankyrin-repeat-deleted form (pKbNAP1Ap), indicating that the antigenic epitopes reside predominantly in the C-terminal domain rather than in the ankyrin extension. Independently, crosslink-dependent immunocapture, the limited effect of low-dose sirtinol on bulk chromosomes and two-dimensional gel analyses indicate that dinoflagellate core histones reside primarily within telomeric anchorages at the nuclear envelope rather than throughout the bulk of birefringent chromosomes [48], a finding directly relevant to interpreting NAP1 localization in relation to the minor histone pool.

In summary, dinoflagellates retain abundant and structurally diverse NAP1 homologs despite low core-histone transcript levels, and their predicted trafficking-signal repertoire is unusually heterogeneous, ranging from dual NLS/NES architectures to the complete absence of either signal class.

### 2.2. Cell-Cycle-Regulated Cytoplasmic and Nuclear Immunofluorescence

**Figure 2:**
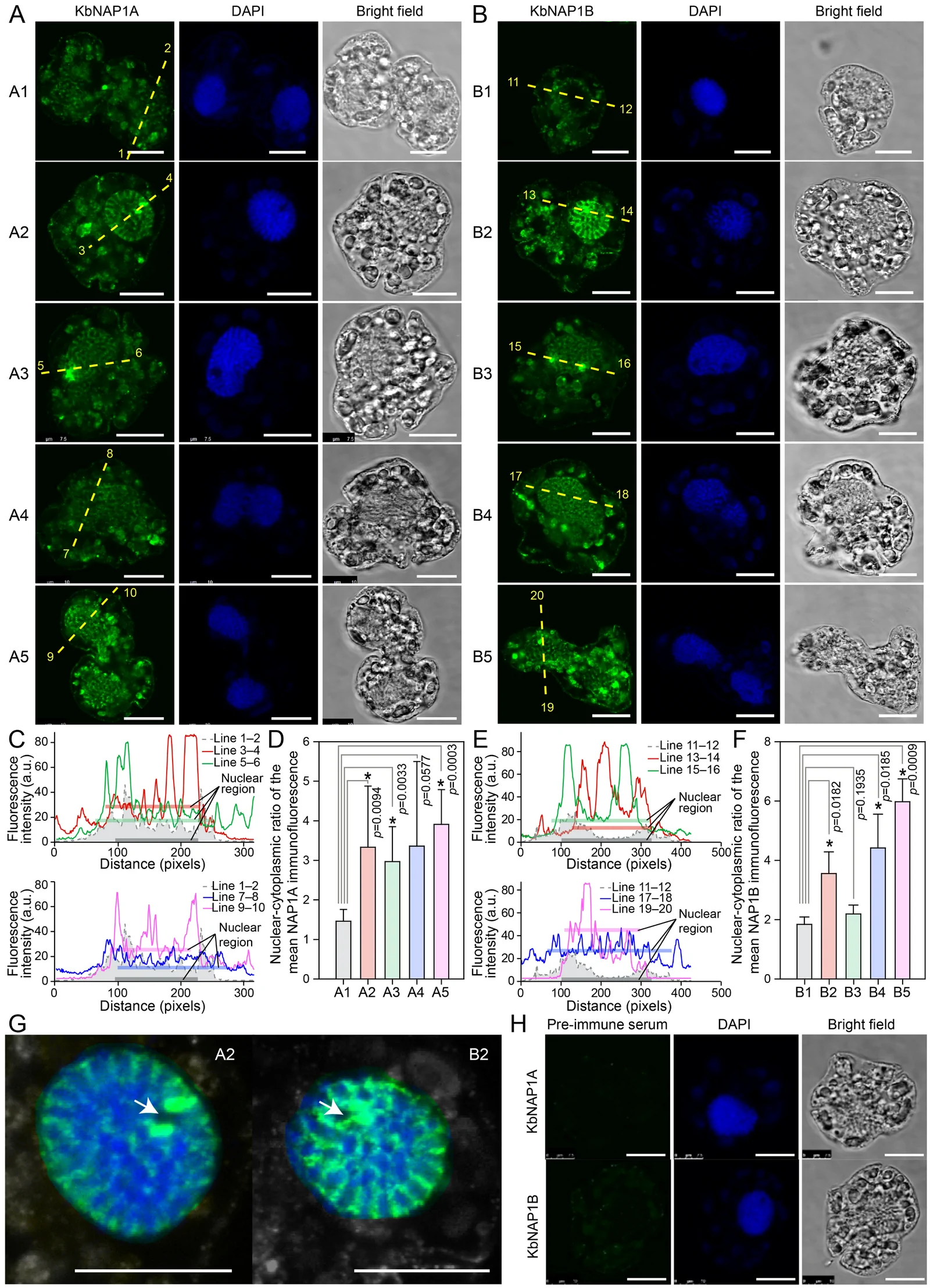
Confocal immunolocalization of KbNAP1A and KbNAP1B in *Karenia brevis* cells at different cell-cycle stages. Confocal immunofluorescence photomicrographs of *K. brevis* cryosections immunostained with (**A**) anti-KbNAP1A or (**B**) anti-KbNAP1B antibody, each with the corresponding DAPI image (**middle column**) and bright field (**right column**). Five representative cell-cycle stages are shown (**A1**–**A5**,**B1**–**B5**), assigned on the basis of nuclear size and morphology: early G_1_ ((**A1**,**B1**), nuclear diameter ~20 µm), late G_1_ (**A2**,**B2**), S-G_2_ with splitting nucleoli (**A3**,**B3**), anaphase (**A4**,**B4**) and post-telophase ((**A5**,**B5**), nuclear diameter ~35 µm). Yellow dashed lines indicate fluorescence intensity transects. (**C**) Fluorescence intensity profiles along transects 1–2, 3–4 and 5–6 (**upper panel**) and 1–2, 7–8, and 9–10 (**lower panel**) from panel (**A**). (**D**) Nuclear-to-cytoplasmic mean immunofluorescence intensity ratios for anti-KbNAP1A-stained cells (**A1**–**A5**). (**E**) Fluorescence intensity profiles along transects 11–12, 13–14 and 15–16 (**upper panel**) and 11–12, 17–18, and 19–20 (**lower panel**) from (**B**). (**F**) Nuclear-to-cytoplasmic ratios for anti-KbNAP1B-stained cells (**B1**–**B5**). Data are presented as means ± SD (n = 20). Asterisks (*) denote statistically significant differences relative to the respective early G_1_ reference cell ((**A1**) or (**B1**); *p* < 0.05); *p*-values are indicated above bars. (**G**) Merged higher-magnification pseudocolor images of representative late-G_1_ cells (**A2**,**B2**), overlaying DAPI (blue) with KbNAP1Ap or KbNAP1Bp (green), illustrating inter-chromosomal localization; white arrows indicate nucleoli. (**H**) Pre-immune serum controls for anti-KbNAP1A (**upper**) and anti-KbNAP1B (**lower**). Nuclear-to-cytoplasmic ratios increased approximately three-fold from early G_1_ (**A1**,**B1**) to post-telophase (**A5**,**B5**), consistent with progressive nuclear accumulation of both homologs during S–G_2_/M in a cell that lacks nuclear envelope breakdown. Nucleolar signal, prominent in (**B3**), was undetectable at anaphase (**B4**). Punctate cytoplasmic staining was observed with both antibodies; no organelle marker was used and no compartment assignment is made. Green, Alexa Fluor 488; blue, DAPI. Scale bars = 10 µm.

NAP1 homologs are broadly interpreted as contributing to nuclear-cytoplasmic maintenance [25]. To localize the *K. brevis* homologs, we used confocal immunofluorescence of athecate cells, which have flattened nuclei and pronounced cell-cycle changes in nuclear and cell size [49]. G_1_ cells showed weak nuclear labeling for both KbNAP1A and KbNAP1B (Figure 2(A1,B1)) and low cytoplasmic signal (Figure 2C,E). By late G_1_, labeling for both homologs was prominent around chromosome peripheries (Figure 2(A2,B2),G), the location of transcriptionally active peripheral regions [50]. Background-normalized intensity profiles (Figure 2C,E) confirmed this distribution. Both antibodies also gave punctate cytoplasmic staining in G_1_ and G_2_ cells. As no organelle marker was used, we therefore make no compartment assignment for this signal.

Both homologs showed the same subtle shift in nuclear pattern with cell-cycle progression, from a circum-chromosomal distribution in G_1_ to a more node-like inter-chromosomal configuration in G_2_; the biochemical divergence between them (Section 2.3 and Section 2.4) was therefore not resolved by imaging. Nucleoli, which in dinoflagellates persist through mitosis as one or two large DAPI-negative domains containing decondensed ribosomal loci [51,52], were strongly labelled and duplicated before mitosis (Figure 2(B3)), with signal falling below detection at anaphase (Figure 2(B4)). Nucleolar labeling of this kind is consistent with the reported role of yeast Nap1 in ribosomal protein chaperoning and nucleolar function [53].

Nuclear-to-cytoplasmic labeling ratios were higher in G_2_/M than in early G_1_ for both homologs (Figure 2(A4,A5,B4,B5),D,F), an increase of approximately three-fold from early G_1_ to post-telophase. Because the nuclear envelope does not break down, this enrichment must be established by transport rather than by mitotic mixing; whether it also serves the charge-related function proposed in Section 3.3 is not tested by these data.

### 2.3. Immunoprecipitation and Post-Translational Modifications

The extensive inter-chromosomal territory localization of both NAP1 homologs (Figure 2G) prompted us to examine their molecular associations. Chromosome positioning is maintained during G_1_ [48], and the NAP1 labeling pattern overlaps the previously characterized dHLP distribution in nucleoli and PCLs [20,54], where transcriptionally active domains are concentrated [55]. Although dHLPs lack histone folds and concentrate their basic residues in the penultimate helix, an arrangement entirely distinct from the H2A:H2B surface preferred by NAP1, their co-localization and high abundance made the association worth testing.

**Figure 3:**
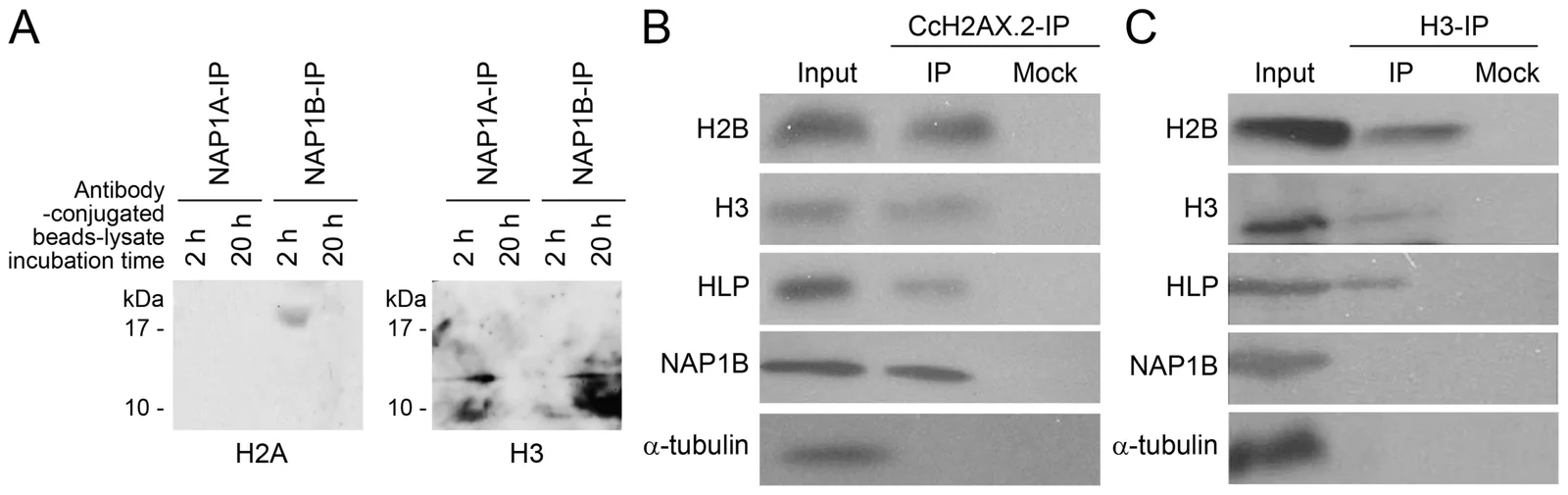
Immunoprecipitation analysis of dNAP1 protein interactions in *Crypthecodinium cohnii*. Immunoblots of (**A**) NAP1-, (**B**) CcH2AX.2- and (**C**) H3-immunoprecipitates from *C. cohnii* total cell lysates. All incubations were performed after lysis; the assay reports differential recovery of immunoreactive material under cell-free conditions and does not establish direct protein–protein interaction, association in the intact cell or interaction kinetics. All antibodies raised in this study were affinity-purified against their respective antigens (Section 4.4). (**A**) Anti-KbNAP1A-IP and anti-KbNAP1B-IP complexes probed for H2A, dHLP and H3 after 2 h or 20 h incubation with cleared lysate. H2A-immunoreactive material was recovered in the anti-KbNAP1B immunoprecipitate at 2 h but not at 20 h, and was not detected in the anti-KbNAP1A immunoprecipitate at either time; loss over prolonged post-lysis incubation is equally consistent with proteolysis, epitope loss or complex dissociation. H3-immunoreactive material was recovered with both antibodies, preferentially at 20 h; gain during prolonged post-lysis incubation is equally consistent with re-association in dilute lysate. (**B**) CcH2AX.2-immunoprecipitates (input, IP and mock) probed for H2B, H3, dHLP, NAP1B and α-tubulin. (**C**) H3-immunoprecipitates probed with the same panel. Input lanes are equally loaded with total lysate before immunoprecipitation. NAP1B-immunoreactive material was recovered in both the anti-CcH2AX.2 and anti-H3 immunoprecipitates but not in mock lanes, consistent with association with CcH2AX.2- and H3-containing chromatin complexes; recovery with two independent antibodies against different histones reduces the likelihood of a single-antibody artefact but does not exclude chromatin-mediated indirectness. dHLPs were likewise recovered in both immunoprecipitates, consistent with their presence in nucleosome-containing chromatin domains. Mock controls used protein A beads alone and therefore exclude non-specific bead binding. Three independent immunoprecipitations gave concordant results.

Before describing the results, we state the limits of the assay, since they bound every inference drawn below. Immunoprecipitations were performed on cleared total cell lysates that had been incubated for 2 or 20 h after lysis; they therefore report complexes recovered under cell-free conditions and cannot establish the kinetics or the existence of an association in the intact cell. Panels B and C include a bead-only mock control, which excludes non-specific binding to protein A beads. The absence of α-tubulin from all IP lanes (Figure 3B,C) shows only that an abundant cytoplasmic protein is not carried through. All antibodies used were affinity-purified against their respective antigens (Section 4.4). We therefore report differential recovery of immunoreactive material between antibodies and between time points and interpret no signal below as a direct protein–protein interaction.

**Figure 4:**
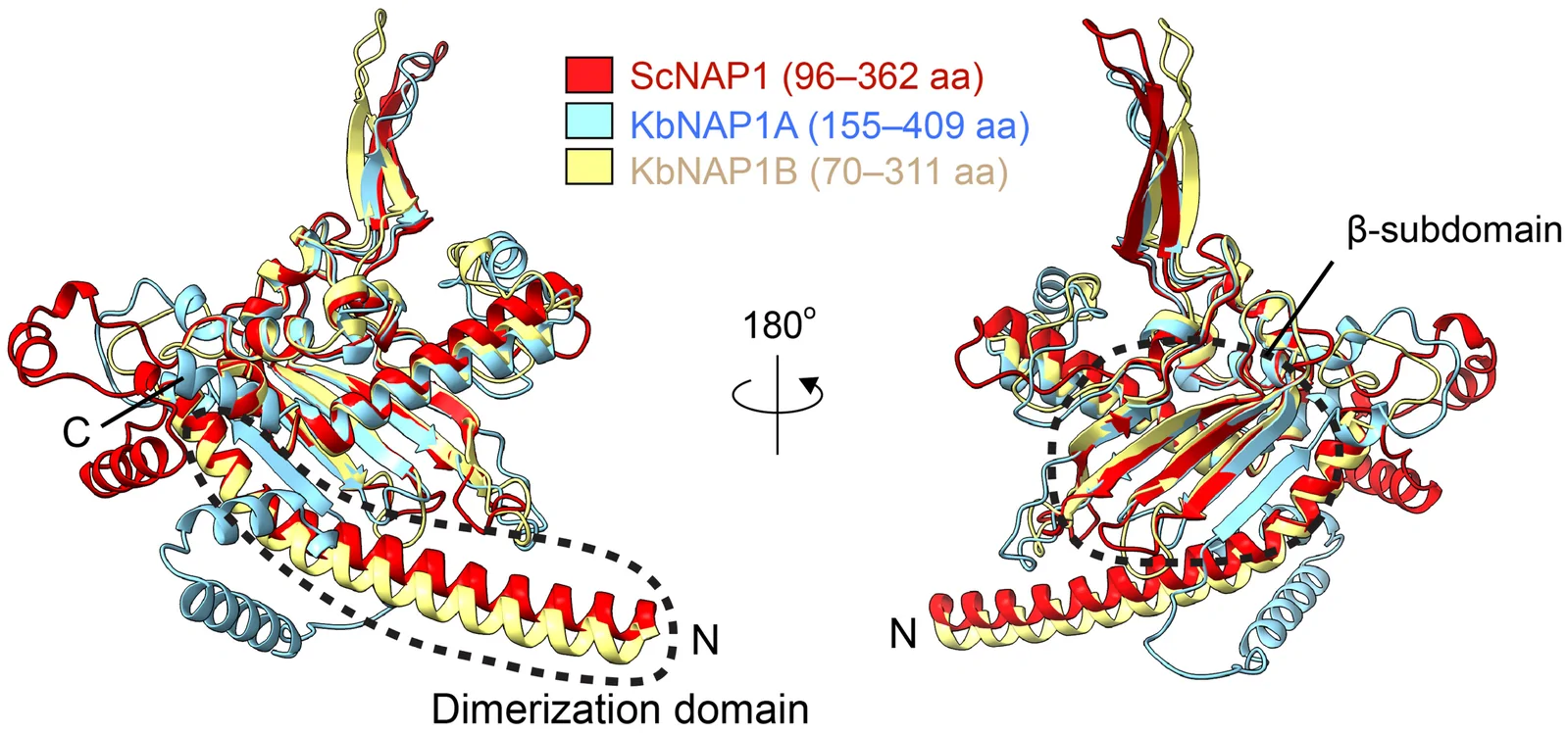
Structural comparison of dinoflagellate and yeast NAP1 domain models. Structural superimposition of the NAP domains of ScNAP1 (red; residues 96–362), KbNAP1A (cyan; residues 155–409) and KbNAP1B (yellow; residues 70–311), generated and aligned as described in Section 4.2.5. The dimerization domain (lower left, dashed box) and β-subdomain (upper right) are indicated. RMSD values (pruned atom pairs/all atom pairs): ScNAP1 vs. KbNAP1A, 0.844 Å/6.943 Å (95/173 pairs); ScNAP1 vs. KbNAP1B, 0.915 Å/5.698 Å (120/228 pairs); KbNAP1A vs. KbNAP1B, 0.692 Å/4.067 Å (120/189 pairs). To place the comparison on an independent, length-normalized and chance-calibrated footing, DALI and TM-align alignments were additionally performed on the same NAP-domain-truncated coordinates (Section 4.2.5). DALI Z-scores, which are calibrated against chance alignments [56], were 21.1 for ScNAP1 vs. KbNAP1B (rmsd 3.4 Å, 218 aligned residues), 15.9 for ScNAP1 vs. KbNAP1A (rmsd 4.0 Å, 168 aligned residues) and 20.2 for KbNAP1A vs. KbNAP1B (rmsd 3.2 Å, 182 aligned residues); TM-align, normalized to the ScNAP1 reference, gave TM-scores of 0.712 and 0.534, respectively. For KbNAP1A vs. KbNAP1B, where no common reference exists, both normalizations are reported (0.821 to KbNAP1A; 0.671 to KbNAP1B); both exceed the 0.5 same-fold threshold. All three metrics rank the two ScNAP1-referenced comparisons concordantly, indicating that KbNAP1B recapitulates the canonical yeast NAP1 fold more closely than KbNAP1A does.

An H2A-immunoreactive band (detected with anti-CcH2AX.2; for *C. cohnii* core-histone identities see Table S1) was recovered in the anti-KbNAP1B immunoprecipitate after 2 h but was not detected at 20 h, and was not detected in the anti-KbNAP1A immunoprecipitate at either time point (Figure 3A). We do not interpret the loss of signal between 2 and 20 h as evidence of a transient chaperone–substrate interaction: loss over prolonged post-lysis incubation is equally consistent with proteolysis, epitope loss or dissociation of the recovered complex, alternatives this experiment cannot distinguish. Because both antibodies were raised against *K. brevis* antigens and cannot resolve individual CcNAP1 paralogs, this is a property of the antibody-reactive material and not of a specified *C. cohnii* protein. The differential is nonetheless in the direction predicted by the structural comparison of Figure 4, in which the β-subdomain of KbNAP1B more closely recapitulates the canonical yeast NAP1 fold; we present this as concordance between two independent lines of evidence obtained in two different organisms, not as identification of the precipitated species.

Anti-CcH2AX.2 and anti-H3 pull-downs gave positive dHLP signals (Figure 3B,C), consistent with the presence of dHLPs in nucleosome-containing chromatin domains. In the same reciprocal pull-downs, NAP1B-immunoreactive material was recovered in both immunoprecipitates but was absent from the mock lanes (Figure 3B,C). Recovery with two independent antibodies directed against different histones reduces the likelihood of a single-antibody artefact, although it does not address chromatin-mediated indirectness; the overall pattern is most simply explained by NAP1 and dHLPs occupying shared chromatin rather than by direct mutual binding. Both anti-KbNAP1A and anti-KbNAP1B recovered H3-immunoreactive material, preferentially at 20 h (Figure 3A). Gain of signal during prolonged post-lysis incubation requires the same caution as loss, since re-association in dilute, uncompartmentalized lysate is a recognized artefact; we therefore do not treat the 20 h preference as physiologically informative, nor as evidence of chaperone activity towards H3. Separately, dinoflagellate nucleosomal domains have been shown to be concentrated at telomeric anchorages at the nuclear envelope rather than distributed throughout birefringent chromosomes [56]; if NAP1–histone associations are similarly restricted, they would be expected to occur predominantly at these minor domains and at select gene loci rather than across bulk chromosomes.

NAP1 homologs are well characterized as carrying multiple PTMs, including N-terminal SUMOylation, C-terminal phosphorylation and ubiquitination, together with concentration-dependent oligomerization [21,44,45]. The number of possible PTM permutations, and of the resulting mass changes, probably exceeds the number of immunoreactive bands observed. The anomalously slow SDS-PAGE migration of highly acidic NAP1 proteins accounts for the baseline offset between predicted and apparent mass even for the unmodified form, whereas the discrete shifts between bands of the same homolog are most parsimoniously explained by PTMs. The strongest direct evidence for post-translational modification is the collapse of the upper immunoreactive bands upon treatment with active, but not heat-inactivated, alkaline phosphatase (Figure S5A), which establishes that the discrete mobility shifts between bands of the same *K. brevis* homolog are phosphorylation-dependent. On this basis the ~72 kDa band of KbNAP1A is consistent in mass with combined phosphorylation and a single ubiquitination (1Ub + 12P; calculated 70.3 kDa; Table S3). We stress that only phosphorylation is directly demonstrated: assignments involving ubiquitination or other modifications are mass-consistency inferences that alternative combinations could also satisfy, and direct confirmation would require mass spectrometry with modification-specific enrichment. No *C. cohnii* band can be assigned to a specific paralog (Section 2.5). The large number of predicted PTM sites is notable for a putative histone chaperone, because in the histone-bound state the dimerized surface would be fully engaged with the H2A:H2B heterodimer; many PTMs may therefore regulate non-chaperone functions or modulate surface charge in non-nucleosomal contexts [21]. FACT, HIRA and additional histone chaperones with chromatin-remodelling activity are also present in dinoflagellate transcriptomes [3], implying some functional redundancy in chromatin maintenance.

High-molecular-weight forms (~150–200 kDa) observed after glutaraldehyde cross-linking (Figure S5B) are suggestive with concentration-dependent dimer-to-tetramer formation, a state more characteristic of free than of histone-loaded NAP1. The ankyrin repeat-deleted pKbNAP1A formed higher-order oligomers more readily than full-length KbNAP1Ap (Figure S5B), implying that the ankyrin repeat domain sterically reduces oligomerization propensity, consistent with the general property of ankyrin repeats to mediate specific protein–protein interactions rather than non-specific self-association [57,58]. Dimerization also modulates unit charge density by differentially exposing charged surfaces, in line with the distinct antigenicity profiles of the two homologs (Figure S6). Finally, the conserved positioning of acidic residues relative to the predicted disordered C-terminal polyglutamine tract (Figure 1 and Figure S1) suggests that these “fishing-line” extensions may adopt different self- and inter-association geometries as a function of ionic strength.

### 2.4. Differential Effects of KbNAP1Ap and KbNAP1Bp on MNase Accessibility of Histone–DNA Complexes and on DNA Electrophoretic Mobility

**Figure 5:**
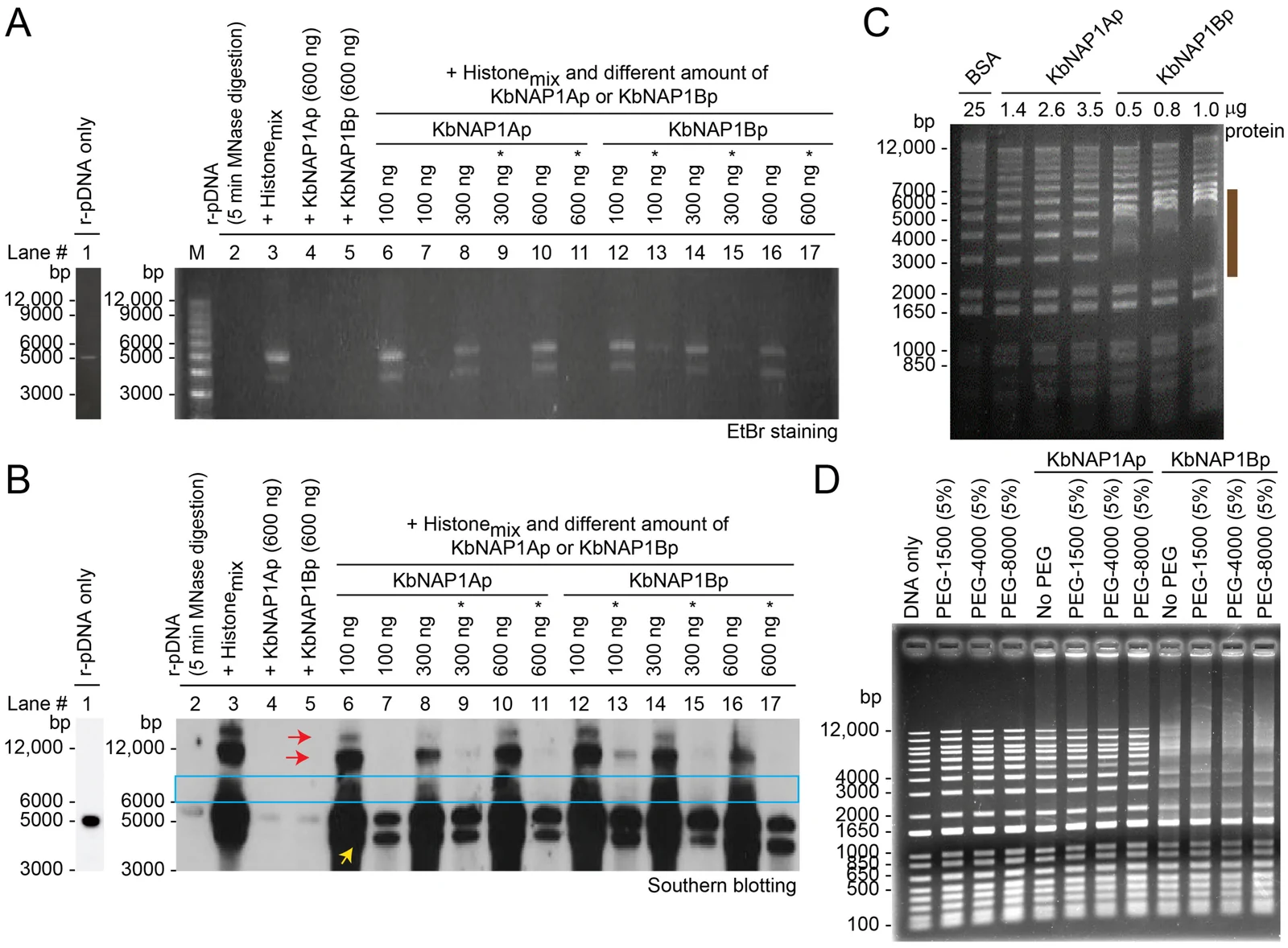
Effects of recombinant KbNAP1Ap and KbNAP1Bp on MNase accessibility of pre-assembled core histone–DNA complexes and on DNA electrophoretic mobility. (**A**,**B**) MNase protection assay of pre-assembled core histone mix (without H1)–relaxed plasmid DNA (r-pDNA) complexes: (**A**) ethidium-bromide-stained 1% agarose gel and (**B**) the corresponding Southern blot (probe: full-length pFastBacHT A, 4.8 kbp). Lanes: L1, r-pDNA alone, no MNase (undigested substrate control); L2, r-pDNA alone, MNase for 5 min (naked-DNA digestion control); L3, r-pDNA + HeLa core histone mix (600 ng), 5 min; L4, r-pDNA + KbNAP1Ap (600 ng), 5 min; L5, r-pDNA + KbNAP1Bp (600 ng), 5 min; L6–L11, r-pDNA + core histone mix (600 ng) + KbNAP1Ap at 100, 300 and 600 ng, each digested for 5 min (L6, L8, L10) or for 15 min (L7, L9, L11); L12–L17, as L6–L11 but with KbNAP1Bp at 100, 300 and 600 ng, digested 5 min (L12, L14, L16) or 15 min (L13, L15, L17). Asterisks denote 15 min digestions. M (in (**A**) only), 1 kb Plus DNA ladder; the equivalent lane in (**B**) is blank because the plasmid probe does not hybridize to the ladder, and the bp scale in (**B**) is transferred from (**A**). L1 was run as a separate gel and blot within the same experimental series, is shown with its own bp scale and separated from L2–L17, was not loaded or exposed comparably with the remaining lanes, and documents only the integrity and electrophoretic mobility of the undigested substrate, not its topological state. In (**B**), red arrows indicate MNase-resistant high-molecular-weight bands (~10–12 kbp) present specifically in L12 (100 ng KbNAP1Bp, 5 min) and absent at higher KbNAP1Bp amounts under the same digestion time (L14, L16); the yellow arrow indicates a concomitant MNase-resistant species at ~3 kbp. The blue box in (**B**) demarcates the ~6–8 kbp window of elevated hybridization signal common to histone-containing lanes (e.g., L3, L6, L8, L10, L12, L14, L16), attributable to protection by the histone mix. Because SDS and proteinase K were applied before electrophoresis, and because linearized and defined-topology plasmid standards were not included, these species are reported as observed, without mechanistic assignment; gels were not quantified densitometrically. Neither homolog produced robust nucleosome assembly or ordered arrays. (**C**) Electrophoretic mobility shift assay. BSA (25 µg ≈ 380 pmol), KbNAP1Ap (1.4/2.6/3.5 µg ≈ 28/52/70 pmol) or KbNAP1Bp (0.5/0.8/1.0 µg ≈ 12/19/23 pmol) was incubated with 1 kb Plus DNA ladder (200 ng per lane) and resolved on a 1% agarose gel (Section 4.3); no separate marker lane was run, and the BSA lane serves as the ladder mobility reference. Molar amounts throughout this figure are calculated from the predicted masses of the deduced untagged sequences (KbNAP1A 49.8 kDa; KbNAP1B 43.2 kDa; Table S2), not from apparent SDS-PAGE mobility, which is inflated for these highly acidic proteins (Section 2.1). KbNAP1Bp reduced the mobility and intensity of higher-molecular-weight fragments in an amount-dependent manner (brown vertical bar at right, ~3–7 kbp), whereas KbNAP1Ap, assayed at higher molar amounts, produced no comparable change; BSA produced no retardation. Ladder fragments differ in sequence composition, molar abundance and potential binding-site number, and are therefore not equivalent competitors; the two proteins were compared over non-overlapping molar ranges, and all complexes were formaldehyde-fixed before electrophoresis. Results are reported as differential ladder-fragment retardation under these specific conditions and are not interpreted as evidence of an intrinsic DNA-length preference. Three independent assays gave concordant patterns. (**D**) Effect of macromolecular crowding. 1 kb Plus DNA ladder (300 ng per lane) was incubated without protein (L1–L4), with KbNAP1Ap (300 ng ≈ 6.0 pmol; L5–L8) or with KbNAP1Bp (300 ng ≈ 6.9 pmol; L9–L12), in the absence of PEG (L1, L5, L9) or with 5% (*w*/*v*) PEG-1500 (L2, L6, L10), PEG-4000 (L3, L7, L11) or PEG-8000 (L4, L8, L12). L1 serves as the ladder mobility reference; L2–L4 control for effects of PEG on mobility and staining in the absence of protein; L5 and L9 provide the no-crowding baseline for each protein. Relative to its own baseline (L9), KbNAP1Bp showed a further reduction in mobility and intensity of the largest fragments (>8 kbp) with PEG, irrespective of PEG molecular weight, whereas KbNAP1Ap showed no comparable change (L5–L8). Three independent assays gave concordant patterns.

Because the anti-KbNAP1B immunoprecipitate recovered H2A-immunoreactive material whereas the anti-KbNAP1A immunoprecipitate did not (Section 2.3), we asked whether either recombinant homolog altered the nuclease accessibility of pre-assembled core histone–DNA complexes. Undigested relaxed plasmid DNA (r-pDNA, 4.8 kbp; Figure 5A,B, L1) documented the integrity and electrophoretic mobility of the starting substrate, and the same substrate was digested by micrococcal nuclease (MNase) in the absence of protein (L2), confirming that unprotected DNA is accessible under these conditions. The two homologs were added on a mass-matched basis (100, 300 and 600 ng), corresponding to ~2.0/6.0/12.0 pmol KbNAP1Ap and ~2.3/6.9/13.9 pmol KbNAP1Bp on the predicted-mass basis used throughout this figure (Section 4.3); panels A and B therefore compare the proteins in approximately equimolar amounts. Addition of KbNAP1Bp (100 ng) was accompanied by MNase-resistant species at ~10–12 kbp and ~3 kbp (Figure 5B, L12) that were absent from the histone-only lane (L3), from every KbNAP1Ap-containing lane (L6–L11) and from 300 and 600 ng KbNAP1Bp (L14, L16). Because SDS and proteinase K were applied before electrophoresis, these species cannot be attributed with confidence to non-covalently protein-bridged plasmid multimers; linearized and defined-topology plasmid standards were not included in this series, and the gels were not quantified densitometrically, so the species are reported as observed, without mechanistic assignment (three independent assays gave concordant band patterns). Neither homolog produced robust nucleosome assembly or ordered arrays, consistent with KbNAP1Bp retaining canonical H2A:H2B-binding capacity without generating extended nucleosomal arrays on plasmid substrates, plausibly because circular DNA does not readily adopt the linker-DNA geometries required for ordered nucleation.

Both paralogs present extensively acidic surfaces, which raises the question of whether they engage DNA at all and, if so, whether they do so differently. We therefore compared them in gel mobility shift assays using a 1 kb Plus DNA ladder as a heterogeneous substrate. KbNAP1Bp reduced the mobility and intensity of higher-molecular-weight fragments in an amount-dependent manner (~3–7 kbp; Figure 5C, brown bar), whereas KbNAP1Ap, assayed at substantially higher molar amounts (~28–70 pmol versus ~12–23 pmol for KbNAP1Bp), produced no comparable change; BSA, in large molar excess (~380 pmol), produced no retardation. Ladder fragments differ in sequence composition, molar abundance and potential binding-site number and are therefore not equivalent competitors; the two proteins were compared over non-overlapping molar ranges; and all complexes were formaldehyde-fixed before electrophoresis. We accordingly report differential retardation of ladder fragments under these specific conditions and do not interpret the result as evidence of an intrinsic DNA-length preference.

We next asked whether the difference between the homologs persisted in the presence of a macromolecular crowding agent. The design is a complete factorial: no protein (L1–L4), KbNAP1Ap (L5–L8) and KbNAP1Bp (L9–L12), each without PEG (L1, L5, L9) and with 5% (*w*/*v*) PEG-1500, PEG-4000 or PEG-8000, so that the effects of PEG on mobility and staining in the absence of protein are controlled directly. Here the two proteins were loaded at equal mass (300 ng), corresponding to approximately equimolar amounts (~6.0 pmol KbNAP1Ap; ~6.9 pmol KbNAP1Bp). Relative to its own no-crowding baseline (L9), KbNAP1Bp showed a further reduction in the mobility and intensity of the largest fragments (>8 kbp) in the presence of PEG, irrespective of PEG molecular weight, whereas KbNAP1Ap showed no comparable change with or without PEG (L5–L8). As in panel C, complexes were formaldehyde-fixed and gels were not quantified densitometrically; these observations are therefore descriptive and are not interpreted as evidence for a volume-depletion or hydrogen-bonding mechanism.

We did not pursue in vitro dHLP-binding assays further. Because NAP1 proteins are strongly acidic and dHLPs strongly basic (Section 1), association between them in dilute buffer is expected on electrostatic grounds and would not be diagnostic of a specific interaction in the cell; consistent with this, the co-precipitation data of Section 2.3 are equally explained by shared chromatin occupancy. We therefore favor, as a working hypothesis (Section 3.3), a role for dinoflagellate NAP1 in the local ionotropic conditioning of the nucleus, for example around the telomeric nucleosomal anchorages before their duplication.

In summary, the two recombinant homologs behaved differently in every in vitro comparison performed: only KbNAP1Bp was associated with altered MNase accessibility of pre-assembled histone–r-pDNA complexes and with retardation of the larger ladder fragments, both in the absence and in the presence of PEG, whereas KbNAP1Ap produced no comparable change even at higher molar amounts. Neither homolog assembled ordered nucleosomal arrays on a circular substrate. The direction of this difference is concordant with the structural comparison of Figure 4 and with the differential recovery of H2A-immunoreactive material in Section 2.3.

### 2.5. Anti-NAP1-Reactive Protein Peaks at S/G_2_, and CcNAP1.1-Antisense-ODN Exposure Is Associated with an S–G_2_/M Delay

Because the MNase and mobility-shift assays indicated that the two recombinant homologs engage histone–DNA and DNA substrates differently, and because NAP1 abundance is cell-cycle regulated in other systems, we examined dNAP1 protein expression across the cell cycle in *C. cohnii*, where highly synchronized G_1_ populations are routinely obtained [59,60,61].

**Figure 6:**
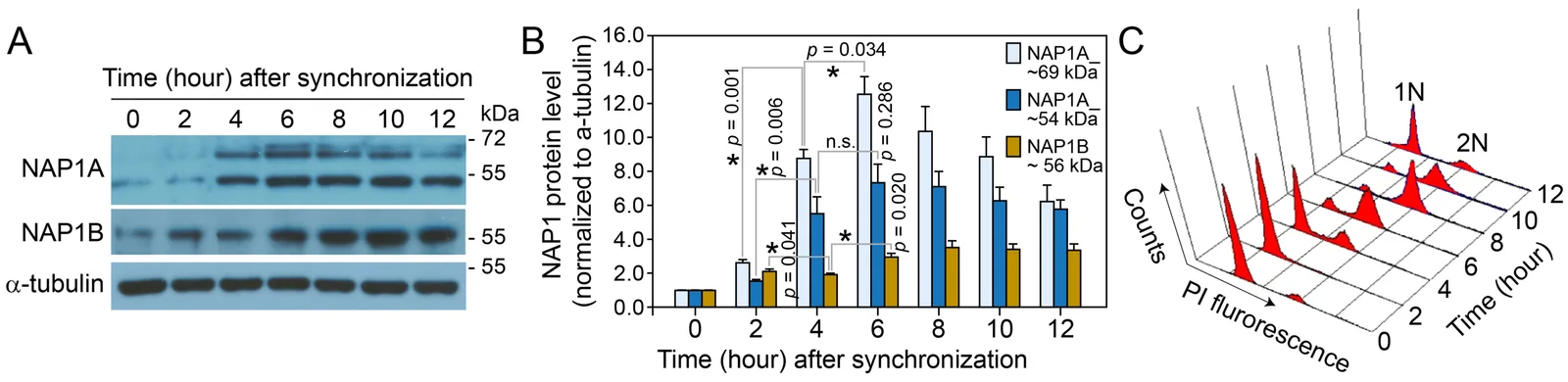
Cell-cycle-regulated abundance of anti-NAP1-reactive protein in synchronized *Crypthecodinium cohnii*. (**A**) Representative immunoblot of anti-KbNAP1A- and anti-KbNAP1B-reactive species in *C. cohnii* lysates harvested at 2 h intervals (T = 0–12 h) after G_1_ swarmer release. Dominant apparent masses were ~69 and ~54 kDa (anti-KbNAP1A-reactive) and ~56 kDa (anti-KbNAP1B-reactive), substantially above the predicted masses of CcNAP1.1 (40.1 kDa), CcNAP1.2 (37.5 kDa) and CcNAP1.3 (38.6 kDa) (Table S3); following the convention of Section 2.1, these species are not assigned to individual paralogs. Equal total protein (50 µg per lane) was loaded and α-tubulin was probed on the same blot as a loading control. Data were cropped from the same blot (no lane removal) with identical exposure settings. The blot is representative of three independent synchronization experiments. (**B**) Densitometric quantification (by ImageJ) of the three antibody-reactive species, each normalized to α-tubulin in the same lane and expressed relative to T = 0 h. Bars show means of n = 3 independent synchronized cultures; error bars indicate SEM. All three species were present at low relative levels during early G_1_ (T = 0–2 rh), rose through T = 4 h, peaked at T = 6 h and declined progressively from T = 8 to 12 h as cells completed mitosis. Statistical comparisons between the time points indicated by brackets were made using an unpaired two-tailed Student’s *t*-test, with exact *p* values shown above each bracket. Asterisks denote *p* < 0.05 and n.s. denotes *p* > 0.05. (**C**) DNA flow cytograms of propidium iodide-stained cells from the same synchronized culture as (**A**), used to assign cell-cycle stage: predominantly 1N (G_1_) at T = 0–4 h, with progressive transition to 2N (S/G_2_/M) from T = 6 h and 2N predominating by T = 8–10 h. Apparent masses varied by ±3–4 kDa between independent gels and are reported relative to the antibody-characterization standards in Figure S4.

**Figure 7:**
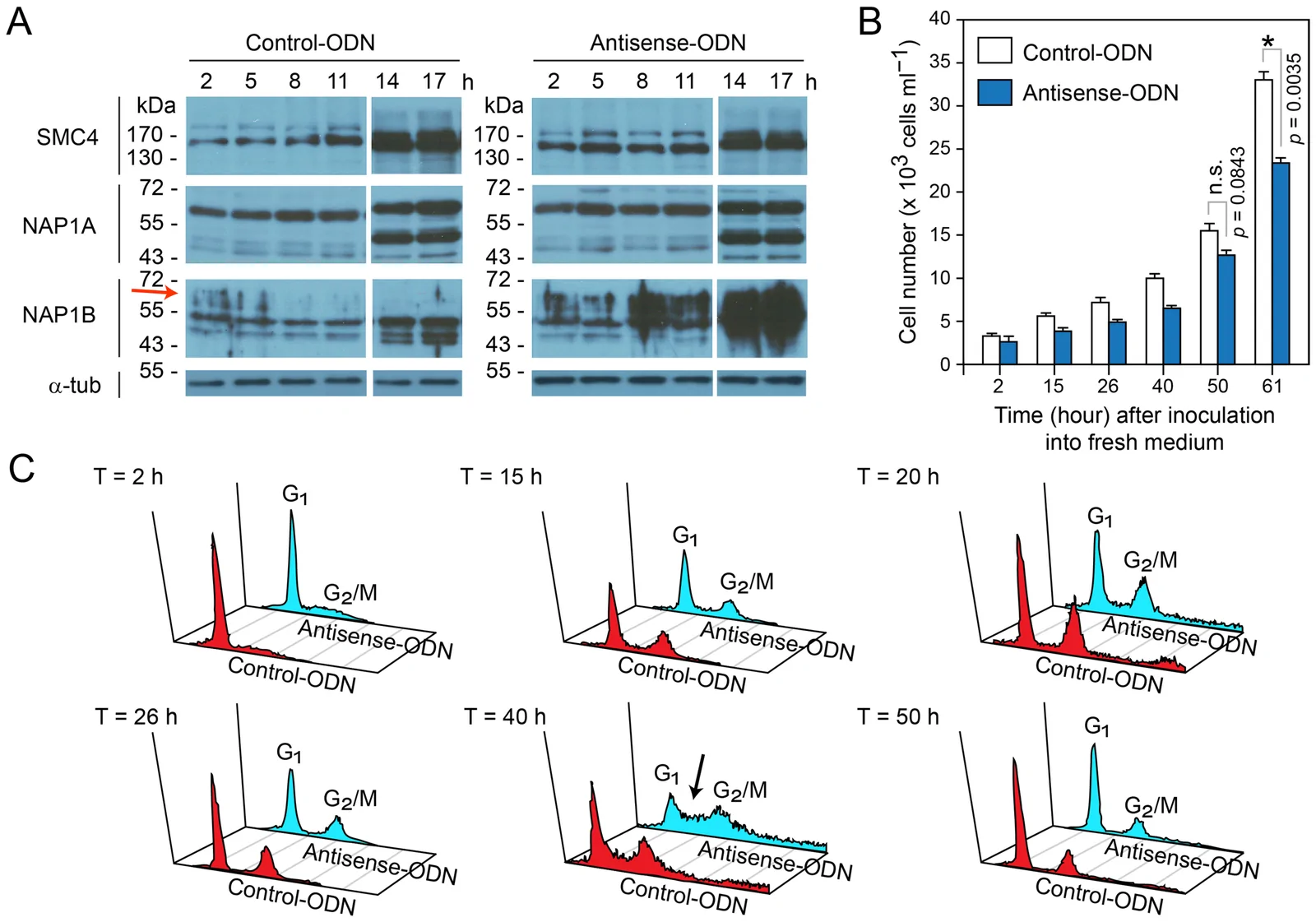
Exposure to *CcNAP1.1* antisense oligodeoxynucleotide is associated with an S–G_2_/M delay in *Crypthecodinium cohnii*. (**A**) Immunoblot analysis of *C. cohnii* cells transfected with control ODN (sense sequence) or *CcNAP1.1*-antisense-ODN (targeting positions 53–73 of the *CcNAP1.1* 5′ UTR). Cells were collected at T = 2, 5, 8, 11, 14 and 17 h, where T = 0 is re-inoculation into fresh medium following transfection. Equal amounts of total protein (50 µg per lane) were loaded. Blots were probed with anti-SMC4 (~170 kDa; condensin subunit; cell-cycle-progression reference, not a loading control), anti-KbNAP1A (the ~69/~54 kDa species migrated at ~65/~55 kDa in this gel), anti-KbNAP1B (detecting species at ~72, ~55 and ~43 kDa), and anti-α-tubulin (~50 kDa; loading control). Following the naming convention of Section 2.5, these are referred to only as anti-KbNAP1A- or anti-KbNAP1B-reactive species and are not assigned to individual CcNAP1 paralogs. The α-tubulin signal was comparable across all time points and between treatments, indicating comparable loading. In control ODN-treated cells, the 72 kDa anti-KbNAP1B-reactive species (red arrow) was detected at T = 2–5 h and was not detected at T = 8–17 h. In *CcNAP1.1*-antisense-ODN-treated cells, it persisted and increased in intensity at T = 8–17 h, a pattern not observed in control ODN-treated cells. The ~72 kDa species may represent a post-translationally modified form or a higher-order assembly; because depletion of the *CcNAP1.1* transcript or its protein product was not directly confirmed, we do not attribute this accumulation to a specific isoform stoichiometry mechanism. A uniform decrease in total NAP1 protein signal was not expected, since both antibodies recognize multiple reactive species, none of which can be assigned to a specific paralog. Blots are representative of three independent transfections; band intensities are compared qualitatively with reference to the α-tubulin signal and total-protein loading. All antibodies were affinity-purified against their respective antigens. (**B**) Cell proliferation curves for control ODN- and CcNAP1.1-antisense-ODN-transfected cells over 61 h. Antisense-ODN treatment was associated with significantly reduced proliferation relative to control from T = 26 h onward (unpaired two-tailed Student’s *t*-test, * *p* < 0.05). Points show the mean of *n* = 3 independent transfections and error bars indicate SD. (**C**) DNA flow cytometric profiles of propidium iodide (PI)-stained *C. cohnii* cells at T = 2, 15, 20, 26, 40 and 50 h, comparing *CcNAP1.1*-antisense-ODN-treated cells (cyan) with sense-control ODN-treated cells (red). G_1_ and G_2_/M peaks are labelled at each time point. Broadening of the inter-peak region, consistent with S–G_2_/M accumulation (black arrow, T = 40 h), was observed in antisense-ODN-treated cells in both independent transfections and in neither control. Normal cell-cycle progression enters S phase approximately 15–17 h after coccoid release.

Both antibodies recognized major bands of ~69, ~54 and ~56 kDa in *C. cohnii* lysates (Figure 6A), substantially above the predicted masses of CcNAP1.1 (40.1 kDa), CcNAP1.2 (37.5 kDa) and CcNAP1.3 (38.6 kDa), consistent with the combined effects of anomalous acidic migration and PTMs (Table S3). Apparent masses varied by ±3–4 kDa between independent gels and between the synchronized-culture (Figure 6) and transfection (Figure 7) experiments; for clarity we refer throughout to the ~69/54 kDa (anti-KbNAP1A-reactive) and ~56 kDa (anti-KbNAP1B-reactive) species, noting that the same modified form may present at a slightly different apparent mass under different running conditions. Because both antibodies were raised against *K. brevis* antigens sharing only 27.8–34.8% identity with the *C. cohnii* isoforms over the immunogen region (Figure S4G,H), we cannot assign these signals to specific CcNAP1 paralogs and describe them only as anti-KbNAP1A- or anti-KbNAP1B-reactive species of the stated apparent mass, in accordance with the convention set out in Section 2.1.

Anti-NAP1-reactive protein was followed across the first synchronized cell cycle by immunoblotting at 2 h intervals after G_1_ swarmer release [62]. All three reactive species were detected throughout the cycle, with significantly lower relative levels (normalized to α-tubulin) during early G_1_ (0–4 h), peaking at S/G_2_ (T = 6 h), followed by a progressive decline from T = 8 to 12 h as cells entered and completed mitosis (Figure 6B,C). At the T = 6 h peak, levels were approximately five- to six-fold higher than at early G_1_ (T = 0–2 h). Upregulation at S–G_2_/M is unexpected for a protein whose canonical function, H2A:H2B shuttling, is tied to replication-dependent histone deposition, particularly as dinoflagellate S phase does not involve wholesale chromosome decondensation but only partial, localized decompaction [62]. One interpretation, developed as an explicit working hypothesis in Section 3.3, is that dNAP1 abundance tracks the increased polyanionic load of genome duplication.

To ask whether CcNAP1.1 contributes to cell-cycle progression, we applied an antisense oligodeoxynucleotide (*CcNAP1.1*-antisense-ODN) directed at the 5′ untranslated region of the *CcNAP1.1* transcript in synchronized *C. cohnii* cells (Figure S8; Section 4.2.7). In control ODN-treated cells, the anti-KbNAP1B blot detected a ~72 kDa immunoreactive species at early time points (T = 2–5 h) that was not detected at T = 8–17 h, indicating cell-cycle-dependent processing or turnover as cells approach S-phase entry (~15–17 h after coccoid release). In *CcNAP1.1*-antisense-ODN-treated cells this species persisted and increased in intensity at T = 8–17 h, a pattern not seen in controls. Because the antibodies do not distinguish the three CcNAP1 paralogs, we do not assign this species to a particular isoform, oligomeric state or processing pathway, and we do not attribute its persistence to a change in isoform stoichiometry. The α-tubulin signal was comparable across time points and treatments, so the persistence is not attributable to unequal loading (Figure 7A).

*CcNAP1.1*-antisense-ODN treatment was accompanied by significantly reduced cell proliferation from T = 26 h onward (Figure 7B) and by a pronounced S–G_2_/M delay, evident as broadening of the inter-peak region in DNA flow-cytometric histograms (Figure 7C, black arrow, T = 40 h). Accumulation of the anti-KbNAP1B-reactive ~72 kDa species (T = 8–17 h), onset of the S-phase delay by flow cytometry (T = 20–26 h) and suppression of proliferation (T = 26 h onward) are temporally ordered. Two limitations bound the interpretation. First, no isoform-specific reagent is available with which to confirm depletion of the CcNAP1.1 product at the protein level; because the intended mechanism is steric interference with the mature transcript rather than nuclease-dependent degradation, transcript abundance is not expected to fall and would not by itself have established target engagement (Section 4.2.7). Reagent specificity is therefore established at the sequence level only, by divergence of the targeted 5′-UTR window from those of *CcNAP1.2* and *CcNAP1.3* (Figure S8). Second, the design includes a sense-orientation control ODN of identical length and composition, but no scrambled ODN, independent antisense sequence or rescue construct; an off-target antisense effect on an unrelated transcript therefore cannot be excluded. We accordingly report an S–G_2_/M delay associated with *CcNAP1.1*-antisense-ODN exposure and make no claim of non-redundancy among the three isoforms. The delay resembles that produced by condensin SMC4 knockdown in the same system [49], compatible with a genome-wide effect on chromosome organization rather than a narrow, substrate-specific histone-chaperoning defect, although the present data do not discriminate between these possibilities.

In summary, in *C. cohnii*, the S/G_2_ peak in anti-NAP1-reactive protein and the S–G_2_/M delay associated with *CcNAP1.1*-antisense-ODN exposure together indicate that dNAP1 abundance and cell-cycle progression are coupled, although these assays do not identify the step at which NAP1 acts. Both observations are consistent with the possibility that dinoflagellate NAP1 proteins function beyond canonical histone chaperoning during genome duplication, a possibility developed as an explicit working hypothesis in Section 3.3.

## 3. Discussion

### 3.1. Histone-Directed Activity Is Partial and Asymmetrically Distributed Between the Two K. brevis Paralogs

The structural and biochemical data indicate that the two recombinant *K. brevis* homologs have diverged in canonical, histone-directed activity. KbNAP1Bp, whose NAP1 domain more closely resembles yeast ScNAP1 by RMSD, DALI Z-score and TM-score (Figure 4), recovers H2A-immunoreactive material in a mock-controlled immunoprecipitation (Section 2.3) and alters MNase accessibility of pre-assembled histone–DNA complexes (Section 2.4), a profile compatible with a residual histone-chaperone role. KbNAP1Ap recovers no H2A-immunoreactive material, forms higher-order oligomers less readily than its ankyrin-deleted form (Figure S5B) and is localized prominently around chromosome peripheries and nuclear cortices at G_1_, a distribution that is more consistent with structural or transport roles than with active histone delivery. The ankyrin-repeat domain of KbNAP1A is also found in the *Brassica* NRP domain, which mediates nuclear localization and complex formation in plant NRP proteins [41], raising the possibility that KbNAP1A represents a functional intermediate between canonical NAP1 and the NRP/SET clade. Because the immunoprecipitations were performed in *C. cohnii* lysates with *K. brevis* antibodies, the concordance between the H2A result and the structural comparison is a cross-species inference, not a demonstration that a particular *C. cohnii* paralog binds H2A.

NAP1 homologs serve multiple roles beyond histone chaperoning, including facilitation of transcription elongation, mRNP biogenesis and PP2A regulation via cohesin dissociation [30,31]. The strong nucleolar immunofluorescence signals and the S-G_2_ increase reported here are consistent with the role of yeast Nap1 in ribosomal-protein chaperoning as part of the chaperOME [53]. Unicellular organisms must respond to environmental change more directly than mammalian cells, and it is notable that ~10% of yeast transcripts are altered in *∆nap1* cells, a phenotypic breadth well beyond that expected of a simple nucleosome assembly factor [63,64,65].

### 3.2. Non-Canonical Roles in the Context of Broader NAP1 Biology

The altered cell-cycle progression associated with *CcNAP1.1*-antisense-ODN exposure is partially consistent with a cell-cycle-modulating role for NAP1, which in this case operates independently of its canonical nucleosome-modulating functions. In *Saccharomyces cerevisiae*, Nap1 participates in the Clb2-Nap1-Gin4 mitotic axis, with *∆nap1* cells showing impaired isotropic bud growth, reduced Gin4 kinase activity and synthetic lethality with *∆cln1∆cln2* [66,67]. The NES-dependent nucleocytoplasmic shuttling of yeast Nap1 is mechanistically inseparable from its mitotic function, since *nap1* mutants lacking the NES fail to complement the mitotic delay of *∆nap1* cells [28]. In *Magnaporthe oryzae*, the MoNap1 NES is required for pathogenicity and appressorium formation, although it is dispensable for cytoplasmic localization per se [65], implying that the dynamics of nucleocytoplasmic cycling, rather than steady-state compartmentalization, carry the functional information. Our ELM screen predicts co-occurring NLS and NES matches in 30.3% of dinoflagellate sequences, including KbNAP1A, CcNAP1.1 and CcNAP1.2 (Section 2.1). By analogy with these systems, we speculate that the regulated redistribution of NAP1 to the nucleus at S/G_2_ (Figure 2D,F and Figure 6), rather than nuclear accumulation per se, may be relevant to the altered cell-cycle profile observed here.

The following motif-based observations are preliminary, sequence-derived predictions from ELM, not experimentally validated functional assignments, and the frequencies quoted are descriptive rather than tests of enrichment (Section 4.2.3). In plants, tobacco NAP1 interacts with tubulin and mitotic cyclins (Nicta;CYCB1;1) [68]. Reverse-orientation Cyclin A2 docking matches (DOC_CYCLIN_RevRxL_6) occur in 51.5% of dinoflagellate sequences and in none of the plant NAP1 sequences examined (Figure S3B). Docking motifs of this class act independently of the kinase catalytic site, anchoring substrates on the cyclin hydrophobic patch and thereby raising local substrate concentration at the kinase. Their frequency in dinoflagellate NAP1, together with the phosphatase-sensitive mobility shifts documented here (Figure S5A; Tables S3 and S4), is compatible with, but does not establish, active tethering of dNAP1 to cyclin–CDK complexes; because CDK phosphorylation adds negative charge, such events would also be expected to modulate the interaction landscape of an already strongly acidic protein. The Rev1 C-terminal domain-binding motif (LIG_REV1ctd_RIR_1) is matched at high frequency in dinoflagellate NAP1 (29/33; 87.9%) compared with human (25.0%) and yeast NAP1 (50.0%) (Figure S3A,B). This motif is normally used by translesion DNA synthesis (TLS) polymerases and accessory factors to dock on Rev1, which recruits TLS machinery to stalled forks [69], so its frequency raises the possibility that NAP1 could act as an accessory chromatin-proximal docking platform, a capacity of potential value given dinoflagellate genome sizes and UV resilience [69]. Caspase-3/7 cleavage-site matches are likewise frequent (CLV_C14_caspase3–7, 75.8% in dinoflagellates versus 28.6% in plants; Figure S3B), which would be consistent with NAP1 turnover being coupled to stress-response proteolysis; because canonical caspases have not been unambiguously characterized in dinoflagellates, which possess metacaspase-family proteases of debated specificity, this link is speculative pending biochemical confirmation. In *Arabidopsis*, NAP1 and NRP proteins act extranucleosomally to promote somatic homologous recombination under genotoxic stress [70]. Whether the *C. cohnii* DNA damage response pathways characterized previously [59] regulate NAP1 abundance, turnover or distribution was not addressed here; testing NAP1 levels and localization under genotoxic challenge, informed by the sequence-level frequencies of Rev1- and caspase-associated motifs noted above, is a logical priority.

Finally, the sensitivity of NAP1 function to its own surface charge is supported by evidence from other systems. For example, Δ*nap1* cells show reduced Gin4 kinase activity and suppress the Δ*swe1* phenotype [66]. Nap1 and Chz1 act redundantly in H2A.Z deposition and restrain heterochromatin formation at promoters in yeast [71], and drought-sensitive rice *ds8* mutants of a NAP1-like gene show altered stomatal function [72], a phenotype mediated through turgor and ion homeostasis that parallels, at least formally, the ionotropic role considered below.

### 3.3. A Chromo-Ionotropic Model for NAP1 Function in the Dinokaryon

Several observations are difficult to reconcile with a purely canonical histone-chaperone role. Anti-NAP1-reactive protein is abundant relative to the small histone pool of the dinokaryon and increases several fold at S/G_2_ (Figure 6B), whereas *CcNAP1.1* antisense-ODN exposure is associated with an altered cell-cycle profile (Figure 7). Moreover, immunolocalization is inter-chromosomal and nucleolar rather than chromosome-body-restricted (Figure 5) and recombinant KbNAP1Bp retards DNA migration under the conditions described in Section 2.4. These observations motivate what we set out explicitly as a working hypothesis, which we term the chromo-ionotropic model, comprising two separable sub-hypotheses: H1, local counterion buffering, and H2, structural partitioning of chromosome territories independently of any buffering function. H1 and H2 are not mutually exclusive, and the present data do not distinguish between them.

Two features of the dinokaryon frame the model. First, S phase does not involve global chromosome decondensation; decompaction appears restricted to local replication and transcription zones, and nascent DNA is accommodated within the volume of the pre-existing condensed chromosome body, the number of nested arches doubling between G_1_ and G_2_ with little change in chromatid diameter [50,62]. Second, the nuclear envelope never breaks down [50,62], so the nucleoplasm is a closed compartment whose protein content is set solely by nucleocytoplasmic transport. The model does not assume that an intact envelope prevents ionic equilibration: nuclear pores freely pass ions and small solutes, and bulk nucleoplasmic ionic strength is expected to equilibrate with the cytoplasm. The constraint we invoke is local: a near-doubling of the amount of polyanion within an approximately conserved volume constitutes an increase in local charge density, arising at dispersed decondensation sites, that is not resettable by the global decompaction or mitotic mixing available to organisms with open mitosis.

The chemistry of the acidic tracts could act on that local environment (H1). NAP1 acidic tails are extended, disordered and functionally distributed, contributing to histone binding synergistically and non-saturably rather than through a defined pocket [73], and therefore lack the densely packed, rigid coordination geometry of an EF-hand site. This matters because selectivity for structural cations, including Ca^2+^ over Mg^2+^, in folded sites is dictated not by electrostatics, which favor the smaller, denser Mg^2+^, but by a many-body polarization penalty imposed by tightly packed carboxylates that falls disproportionately on Mg^2+^ [74]. A flexible, low-packing-density acidic tract should impose a much smaller penalty, should be comparatively permissive to both cations, and could engage Mg^2+^ through solvent-shared, partially hydrated coordination without paying a full desolvation cost. That one protein can host both ions at physiological concentrations is established for cardiac troponin C, where Mg^2+^ occupies the structural sites and competes measurably at the regulatory site [75], and for calmodulin, where Mg^2+^ binds at sites distinct from the EF-hand Ca^2+^ sites [76]. The prediction for NAP1 is therefore many weak sites of high aggregate capacity: a buffer, not a switch. We do not propose that an acidic protein neutralizes DNA charge, which would be self-contradictory; the proposed function is modulation of local free divalent-cation activity, not of net charge.

This could be consequential in a nucleus where DNA carries ~0.6 divalent cations per base pair at a Mg^2+^:Ca^2+^ ratio near 3:1 [35], and where isolated chromosomes require both Ca^2+^ and Mg^2+^ for stability [34]. A mobile, high-capacity acidic reservoir concentrated between chromosome territories could damp local swings in free Mg^2+^ and Ca^2+^ at decondensation zones, so that the surrounding chromosome body remains condensed while replication proceeds, while nuclear accumulation of NAP1 at S/G_2_ would supply that capacity where and when it is required. Phosphorylation offers an obvious tuning variable, since it adds charge to the same tracts and, in yeast, CK2-site mutants show prolonged S phase and altered nuclear import [39]. We note only that the persistent ~72 kDa anti-KbNAP1B-reactive species is associated with the S–G_2_/M delay (Figure 7A) and that discrete mobility shifts among *K. brevis* NAP1 species are phosphatase-sensitive (Figure S5A; Table S3); whether the *C. cohnii* species is a phospho-form, and whether cation engagement differs between phospho-states, is untested. The apparent increase in the immunoreactive species indicated disassembly of the oligomer to monomer, concomitant with local ionotropic changes.

Independently of buffering (H2), the outward-facing acidic surfaces of dNAP1p could support higher-order chains that demarcate territory boundaries sterically or electrostatically, consistent with the inter-chromosomal signal of Figure 2G, although the oligomeric state in vivo was not determined. Three alternatives remain open: redistribution of existing Mg^2+^ and Ca^2+^ [34,35,37] could accommodate the added polyanion without protein involvement; the elevated S/G_2_ abundance is equally compatible with expanded but canonical chaperone demand, including for dHLPs or DVNPs; and the nucleolar signal may simply reflect ribosome biogenesis [53]. Nor does the model displace the histone-directed activity of KbNAP1Bp; it proposes that two activities are separable, with chaperoning concentrated at the minor telomeric nucleosomal domains and cation buffering operating across the chromosome territories.

The model is testable and currently untested. It predicts (i) that recombinant KbNAP1p binds Mg^2+^ and Ca^2+^ with millimolar-range affinity and high stoichiometry, abolished by acidic-tail truncation; (ii) that the DNA retardation of Figure 5C,D is divalent-cation dependent and that NAP1 shifts the cation threshold for DNA condensation in vitro; (iii) that CK2 phosphorylation alters cation capacity; (iv) that added NAP1 destabilizes isolated dinokaryotic chromosomes at physiological cation concentrations in a tail-dependent manner; and (v) that any effect is detectable as local cation redistribution near decondensation zones, by ion-probe mass spectrometry as in [35], rather than as a change in bulk nuclear ionic strength. No charge-balance, counterion-binding or nuclear ionic-activity measurements have been performed here, and we therefore make no claim of quantitative consistency with any specific buffering requirement; the model should be regarded as a framework for hypothesis testing rather than a demonstrated mechanism.

## 4. Materials and Methods

### 4.1. Dinoflagellate Cell Cultures and Flow Cytometry

*Karenia brevis* (CCMP 2229) was cultured at 18 °C in f/2 medium. Athecate *K. brevis* cells are flattened, with cell-cycle-associated increases in nuclear and cell size that permit visual identification of G_1_ from G_2_ cells (~20 µm to ~35 µm nuclear diameter [49]).

*Crypthecodinium cohnii* (strain 1649; Culture Collection of Algae, University of Texas at Austin) was maintained at 28 °C in standard minimum medium [77] without diel light–dark cycles. Highly synchronized G_1_ cells were obtained by the colony release-filtration method [61]. Cells were grown to 10^5^–10^6^ cells mL^−1^ for biochemical preparations. Because dinoflagellate cells contain very low core-histone concentrations [3,4], relatively large protein loads are required; total protein was quantified (Bradford assay, Bio-rad, Hercules, CA, USA) and equal amounts (50 µg per lane) were loaded for all immunoblot comparisons, with α-tubulin used as an independent confirmation of comparable loading (Section 4.4).

Flow cytometry was performed as described [78]. Events were pre-gated in FSC/SSC dot plots before acquisition of propidium iodide fluorescence to exclude debris and doublets.

### 4.2. In Silico Analyses and Molecular Techniques

#### 4.2.1. Sequence Retrieval and Dataset Composition

Dinoflagellate NAP1 and NAP1-related sequences were retrieved from the NCBI Transcriptome Shotgun Assembly (TSA) database (accessed 14 June 2026) by tblastn using ScNAP1 (UniProt P25293), KbNAP1A and KbNAP1B as queries, with an E-value threshold of 1 × 10^−20^. Hits were retained only if InterProScan 5.78–109.0 reported a significant match to the NAP domain (Pfam PF00956; InterPro IPR002164) using Pfam’s curated model-specific gathering thresholds and default post-processing, as applied by InterProScan without user-defined cut-offs, yielding a final set of 33 dinoflagellate sequences. Twenty comparator sequences were included: four human, four fungal (including *S. cerevisiae*), seven plant NAP1-family proteins and five NAP1-related proteins (plant NRP and yeast Vps75). Accessions for the sequences shown in Figure 1, Figures S1 and S2 are listed in Section 4.7; per-sequence accessions for the full set of 53 are given in Figure S2.

#### 4.2.2. Domain, Physicochemical and Secondary-Structure Analyses

Conserved domains were analyzed with the NCBI Conserved Domain Server; Pfam boundaries were assigned independently with InterProScan (Section 4.2.1). Predicted isoelectric points and molecular weights were calculated with ExPASy ProtParam (https://web.expasy.org/protparam/) (accessed 28 August 2026), and net charge at pH 7 with the Prot Pi protein tool (https://www.protpi.ch/Calculator/ProteinTool) (accessed 28 August 2026). Multiple sequence alignments for Figure S1 were generated with ClustalW in BioEdit v7.7.1.0 [79]. Pairwise identity and similarity of the antigen regions to the *C. cohnii* isoforms (Figure S4G,H) were calculated with SIAS (BLOSUM62; gap open 10, gap extend 0.5). Pairwise identity and similarity of the dinoflagellate homologs to human NAP1L1 (Section 2.1) were calculated in Geneious Prime 2026.1.2 from a MUSCLE 5.1 alignment (PPP algorithm). Secondary structure was predicted with PredictProtein (http://ppopen.rostlab.org) (accessed 28 January 2025), and PTM sites with MusiteDeep (https://www.musite.net/) (accessed 28 January 2025) at a threshold of 0.5 [80]; predicted sites and their 11-residue sequence contexts are listed in Table S4 and should not be interpreted as validated modifications.

#### 4.2.3. NLS, NES and ELM Motif Analysis

Classical monopartite and bipartite NLSs were predicted with cNLS Mapper (cut-off score 5.0; search region, entire sequence) [81]. NESs were additionally assigned by comparison with characterized NES sequences [28,40]. Extended NLS/NES class analysis across the 33 dinoflagellate and 20 comparator sequences (Section 4.2.1) used the ELM resource (http://elm.eu.org), interrogating TRG_NLS_MonoExtN_4, TRG_NLS_MonoExtC_3, TRG_NLS_MonoCore_2, TRG_NLS_Bipartite_1, TRG_NES_CRM1_1 and TRG_NESrev_CRM1_2 [46]. Searches were run without cell-compartment or taxonomic context filters, with the motif probability cut-off set to 100. ELM occurrences are reported as raw presence/absence counts within these defined sets (Figures S2 and S3). They were not tested against a randomized or composition-matched background model, nor corrected for phylogenetic non-independence among the dinoflagellate sequences, several of which are paralogs from the same or closely related genera. All percentages are therefore descriptive frequencies specific to the sets analyzed and should not be read as statistically validated enrichment. Denominators for each taxonomic group are given in the legend to Figure S3B.

#### 4.2.4. Disorder, Hydropathy and Antigenicity Predictions

Intrinsically disordered regions were predicted with AIUPred v2 (web server v1.2.2; accessed 14 June 2026) in “AIUPred—only disorder” mode with default smoothing; residues scoring > 0.5 were classified as disordered [82]. Hydropathy was analyzed with ProtScale (Kyte–Doolittle, nine-residue window) [83]. Antigenicity was predicted with EMBOSS *antigenic* (Kolaskar–Tongaonkar) [84].

#### 4.2.5. Structure Prediction and Structural Comparison

Models were generated with ColabFold v1.5.2 (AlphaFold2 with MMseqs2; msa_mode = mmseqs2_uniref_env, pair_mode = unpaired_paired; accessed 9 April 2023). Dinoflagellate models were aligned to the *S. cerevisiae* NAP1 structure (P25293, AlphaFold Protein Structure Database) with the Matchmaker tool in UCSF ChimeraX v1.2, and RMSD was computed for pruned and for all atom pairs [85]. Pairwise structural alignments were additionally performed with the DALI server [86] and TM-align [87] (both accessed 14 August 2026) on the same NAP-domain-truncated coordinate files used for Matchmaker (Pfam PF00956 boundaries: ScNAP1 residues 96–362; KbNAP1A residues 155–409, N-terminal ankyrin repeats excluded; KbNAP1B residues 70–311. DALI Z-scores and TM-scores (normalized to the ScNAP1 reference for the ScNAP1-referenced comparisons, and to both structures for the KbNAP1A–KbNAP1B comparison) were used as length-independent, chance-calibrated complements to the RMSD values in Figure 4.

#### 4.2.6. Cloning and General Molecular Techniques

Full-length KbNAP1A and KbNAP1B sequences were assembled from partial GenBank TSA contigs, with 5′ and 3′ extensions cloned by nested PCR using a dinoflagellate mRNA spliced-leader (CSL) forward primer and an oligo-dT adaptor. All primer sequences are listed in Table S5. General molecular and cell biological techniques followed published protocols [45,78,88].

#### 4.2.7. Antisense Oligodeoxynucleotide Knockdown

Antisense-ODN-based knockdown in *C. cohnii* used a lipofection–spheroplast transfection protocol [49,88] with Lipofectamine 2000 (Invitrogen) according to the manufacturer’s instructions. The antisense-ODN (5′-GCAAGATGCTAACGTTGAAGG-3′), targeting positions 53–73 of the 112-bp 5′ UTR of the *CcNAP1.1* transcript, was designed with the aid of centroid-based mRNA secondary-structure prediction [89]. The ODN is a single-stranded DNA oligomer and the intended mechanism is steric interference with the mature transcript rather than nuclease-dependent degradation; target transcript abundance is therefore not expected to fall, and RT-qPCR would not by itself establish target engagement. This consideration is reinforced by the strongly post-transcriptional character of dinoflagellate gene regulation, in which protein levels frequently vary while transcript levels remain stable [90], a property linked to the organization of highly expressed genes in tandem arrays [91,92]. Reagent specificity is established at the sequence level by divergence of the targeted 5′-UTR window from the corresponding CcNAP1.2 and CcNAP1.3 5′-UTRs (~23% and ~38% identity; Figure S8). A control ODN of identical length and composition encoding the complementary (sense) sequence, which cannot engage the endogenous transcript by an antisense mechanism, was used for mock transfection. ).

### 4.3. Biochemical Assays

Nucleosome assembly and MNase protection. Relaxed plasmid DNA (r-pDNA; 100 ng per reaction; pFastBacHT A, Invitrogen, 4.8 kbp) was prepared by topoisomerase I treatment (37 °C, 30 min). Pre-assembled nucleosomes were formed by combining r-pDNA with a commercial HeLa-derived histone octamer mix (NEB) in assembly buffer (10 mM HEPES pH 7.5, 50 mM NaCl, 1 mM EDTA, 1 mM DTT) using step-salt dilution from 2 M to 0.1 M NaCl. Recombinant NAP1 proteins (100, 300 or 600 ng) were added and incubated at 30 °C for 30 min. MNase digestion was performed at 37 °C for 5 min (or 15 min as indicated) with 2 mM CaCl_2_ and terminated with Tris-buffered proteinase K (20 mM Tris pH 8.0, 0.5% SDS, 5 mM EDTA, 0.1 mg mL^−1^ proteinase K) at 56 °C for 30 min. Products were resolved on 1% agarose gels and confirmed by Southern blotting with a full-length pFastBacHT A probe. An undigested r-pDNA control (no MNase) was run on a separate gel and blot within the same experimental series and is shown as L1 of Figure 5A,B; it was not loaded or exposed comparably with L2–L17. Linearized and defined-topology plasmid standards were not included, and MNase gels were not quantified densitometrically.

Electrophoretic mobility shift assay (EMSA). For amount-dependent DNA-binding assays (Figure 5C), BSA (25 µg; negative control), KbNAP1Ap (1.4, 2.6 or 3.5 µg) or KbNAP1Bp (0.5, 0.8 or 1.0 µg) was incubated with 1 kb Plus DNA ladder (200 ng per lane) in 20 mM Tris pH 8.0, 100 mM NaCl, 1 mM DTT for 30 min at 22 °C. For macromolecular crowding assays (Figure 5D), KbNAP1Ap (300 ng) or KbNAP1Bp (300 ng) was incubated with 1 kb Plus DNA ladder (300 ng per lane) under the same buffer conditions in the absence or presence of 5% (*w*/*v*) PEG-1500, PEG-4000 or PEG-8000, with no-protein lanes for every PEG condition. All EMSA samples were cross-linked with 0.75% (*w*/*v*) formaldehyde for 30 min at 22 °C and resolved on 1% agarose gels in TAE. Band intensities were not quantified densitometrically.

Glutaraldehyde cross-linking for oligomerization analysis. Recombinant KbNAP1Ap, pKbNAP1Ap (ankyrin-repeat deleted) and KbNAP1Bp were incubated with 0.001%, 0.01% or 0.1% (*w*/*v*) glutaraldehyde in PBS pH 7.4 for 30 min at 22 °C, quenched with 50 mM glycine, resolved by SDS-PAGE and visualized by silver staining (Figure S5B).

Alkaline phosphatase treatment. Total *K. brevis* lysates were treated with active or heat-inactivated (95 °C, 10 min) calf intestinal alkaline phosphatase (CIP; NEB) at 37 °C for 30 min in CIP buffer, then resolved by SDS-PAGE and immunoblotted with anti-KbNAP1A and anti-KbNAP1B (Figure S5A).

Molar amounts. Molar amounts of recombinant proteins are reported throughout on the basis of the predicted masses of the deduced untagged sequences (ExPASy ProtParam; KbNAP1A 49.8 kDa, KbNAP1B 43.2 kDa; Table S2) rather than apparent SDS-PAGE mobility, which is anomalously slow for these highly acidic proteins.

### 4.4. Antibodies and Immunological Techniques

Anti-KbNAP1A (recognizing residues 145–441, excluding the ankyrin repeat) and anti-KbNAP1B antibodies were raised against purified recombinant proteins expressed in *E. coli* SG13009 and purified and refolded according to the QIAexpressionist protocol (Qiagen, Venlo, The Netherlands). Native-like folding of the refolded recombinant proteins was supported by their concentration-dependent, ankyrin-modulated oligomerization (Figure S5B).

Every antibody generated in this study, anti-KbNAP1A, anti-KbNAP1B, anti-CcH2AX.2 and anti-dHLP, was affinity-purified against its membrane-bound antigen [93] after pre-clearing with dried bacterial acetone powder [94]; only affinity-purified preparations were used in the immunoblot, immunoprecipitation and immunofluorescence experiments reported here. Anti-KbNAP1A was additionally pre-incubated with *E. coli* lysate to deplete any anti-ankyrin-repeat reactivity arising from the abundance of ankyrin repeats in the bacterial proteome. Secondary antibodies were pre-cleared with dried dinoflagellate (*C. cohnii* or *K. brevis*) acetone powder extracts [94]. Anti-KbNAP1A did not cross-react with KbNAP1Bp (Figure S4), a specificity confirmed by antibody pre-depletion with the heterologous full-length recombinant protein (Figure S7).

Polyclonal anti-CcH2AX.2 was generated against the C-terminal region of CcH2AX.2 (residues 93–200, including the histone-fold domain; accession MN889535) shared with CcH2AX.1 and recognized both free and nucleosome-associated CcH2A. Polyclonal anti-dHLP was raised against full-length bacterially expressed CcHCc3p and cross-reacts with other dHLP isotypes by virtue of high sequence homology [19]. Commercial antibodies were anti-histone H2B (sc-10808), anti-histone H3 (sc-8645R) and anti-α-tubulin (sc-53646; all Santa Cruz). Lysate preparation and immunoblotting followed published protocols [45,95]; equal total protein (50 µg per lane; Section 4.1) was loaded, α-tubulin was probed on the same blots as an independent confirmation of comparable loading, and band intensities were quantified with ImageJ v1.54g [96]. Anti-SMC4 was used as a cell-cycle-progression reference, not as a loading control, since SMC4 is itself cell-cycle regulated.

Immunoprecipitation followed using established protocols [78]. Mock immunoprecipitation with protein A beads alone excluded non-specific bead binding.

### 4.5. Immunofluorescence Microscopy

Immuno-labeling of whole cells and cryosections followed established protocols [88]. Cryosections (5 µm) were prepared as described [97], with stepwise polyvinylpyrrolidone/sucrose (20% *w*/*v* PVP, 1.7 M sucrose) infiltration [98]. Confocal imaging of anti-KbNAP1A- and anti-KbNAP1B-labelled cryosections with DAPI counterstaining was performed on a Leica TCS SP5 II system, using Alexa Fluor 488-conjugated goat anti-rabbit IgG (green channel). Pre-immune serum from the same rabbits, processed identically, served as the negative control (Figure 2H).

For intensity comparisons, all images were acquired with identical parameters and converted to 8 bit grayscale in ImageJ [96]. Mean gray values and integrated density were measured within ROIs defined by the DAPI signal. Background fluorescence was measured in blank areas and subtracted (total nuclear fluorescence = integrated density − [ROI area × mean background]), and values were normalized to the total nuclear fluorescence of the A1 reference cell (early G_1_). Fluorescence-intensity transects are plotted as distance (pixels) versus fluorescence intensity (a.u.).

### 4.6. Statistical Analyses

All experiments were conducted in at least three biological replicates. Unpaired two-tailed t-tests were performed using GraphPad Prism v8.02. Results were considered statistically significant at *p* < 0.05. Data are presented as means ± SD with n > 3 unless otherwise stated. Gel and blot images in Figure 3 and Figure 5 were not quantified densitometrically; for these, concordance of band patterns across three independent experiments is reported qualitatively.

### 4.7. Accession Numbers

NAP1 proteins: *Karenia brevis* KbNAP1A (MT489705), KbNAP1B (MT489706); *Crypthecodinium cohnii* CcNAP1.1 (MT489707), CcNAP1.2 (MT489708), CcNAP1.3 (MT489709); *Karenia mikimotoi* NAP1A (GHKS01148439.1), NAP1B (GHKS01077379.1); *Symbiodinium* sp. clade C (GBSC01003830.1); *Symbiodinium* sp. clade D (GAFP01007195.1); *Alexandrium tamarense* (GAJG01065758.1); *Noctiluca scintillans* (GELK01049667.1); *Perkinsus marinus* (XP_002764795.1); *Plasmodium falciparum* (pfNAPL: KOB63340.1, pfNAPs: XP_001352061.1); *Paramecium tetraurelia* (XP_001444376.1); *Tetrahymena thermophila* (XP_001022132.2); *Toxoplasma gondii* (XP_002366900.1); *Arabidopsis thaliana* (NP_179538.1); *Phytophthora infestans* (XP_002905283.1); *S. cerevisiae* (CAA82125.1); *Xenopus laevis* (Q7ZY81.1); *Homo sapiens* (NP_004528.1). *C. cohnii* core histones: H2AZ (MN889533), H2AX.1 (MN889534), H2AX.2 (MN889535), H2B.1–H2B.3 (MN889536–MN889538), H3.1–H3.6 (MT956610–MT956615), H4.1–H4.2 (MT956616–MT956617).

## 5. Conclusions

Dinoflagellates retain abundant, cell-cycle-regulated NAP1 homologs despite the near-total loss of an architectural nucleosomal substrate. In *C. cohnii*, anti-NAP1-reactive protein rises several-fold at S/G_2_, and CcNAP1.1-antisense-ODN exposure is accompanied by an S–G_2_/M delay and reduced proliferation; in *K. brevis*, both homologs accumulate in the nucleus from early G_1_ to post-telophase and localize between chromosomes and at nucleoli. The two *K. brevis* homologs diverge in vitro: KbNAP1Bp reproduces the canonical yeast NAP1 fold more closely, recovers H2A-immunoreactive material, alters MNase accessibility of pre-assembled histone–DNA complexes and retards larger DNA fragments in mobility-shift assays, whereas KbNAP1Ap does none of these under the conditions tested. Neither homolog generated ordered nucleosomal arrays, indicating that any residual histone-directed activity is partial and asymmetrically distributed between paralogs. Together, these findings uncouple NAP1 abundance from nucleosome availability and motivate the chromo-ionotropic model as an hypothesis, with local counterion modulation (H1) and structural partitioning of chromosome territories (H2) as separable sub-hypotheses.

## Data Availability

The data presented in this study are openly available in the GenBank database, and their accession numbers are listed in Section 4.7 (Accession Numbers).

## Supplementary Materials

The following supporting information can be downloaded at: https://www.mdpi.com/article/10.3390/ijms27177931/s1.

## Abbreviations

The following abbreviations are used in this manuscript:

Ac	Acetylation
CDK	Cyclin-Dependent Kinase
CIP	Calf Intestinal Phosphatase
CK2	Casein Kinase 2
CRM1	Chromosome Region Maintenance 1 (exportin 1)
CSL	Dinoflagellate mRNA spliced-leader sequence
DAPI	4′,6-diamidino-2-phenylindole
dHLP	Dinoflagellate Histone-Like Protein
dNAP1	Dinoflagellate nucleosome assembly protein 1
ELM	Eukaryotic Linear Motif
EMSA	Electrophoretic Mobility Shift Assay
FACT	Facilitates Chromatin Transcription
FPKM	Fragments per kilobase of transcript per million mapped reads
HIRA	Histone Regulator A
IDR	Intrinsically disordered region
IP	Immunoprecipitation
Me	Methylation
MNase	Micrococcal nuclease
mRNP	Messenger Ribonucleoprotein
MW	Molecular weight
NAP1	Nucleosome assembly protein 1
NES	Nuclear export signal
NLS	Nuclear localization signal
NRP	NAP1-related protein
ODN	Oligodeoxynucleotide
P	Phosphorylation
PCL	Peripheral chromatin loop
PEG	Polyethylene glycol
pI	Isoelectric point
PI	Propidium iodide
PP2A	Protein phosphatase 2A
PTM	Post-translational modification
PVP	Polyvinylpyrrolidone
r-pDNA	Relaxed plasmid DNA
RMSD	Root Mean Square Deviation
ROI	Region of Interest
SDS-PAGE	Sodium Dodecyl Sulfate–Polyacrylamide Gel Electrophoresis
SUMO/Sumo	Small Ubiquitin-like Modifier/Sumoylation
TOPOI	Topoisomerase I
TSA	Transcriptome Shotgun Assembly
Ub	Ubiquitination
UTR	Untranslated region
