# Supplementary material for: Nucleosome Assembly Protein 1 in Dinoflagellate Cells Without Architectural Nucleosomes
Source: Int J Mol Sci. 2026 Sep 5;27(17):7931. doi: 10.3390/ijms27177931 (PMC13565876; doi:10.3390/ijms27177931)
Supplement: Supplementary file 1 [file ijms-27-07931-s001.zip › ijms-4451049-supplementary.pdf]

Supplementary Materials

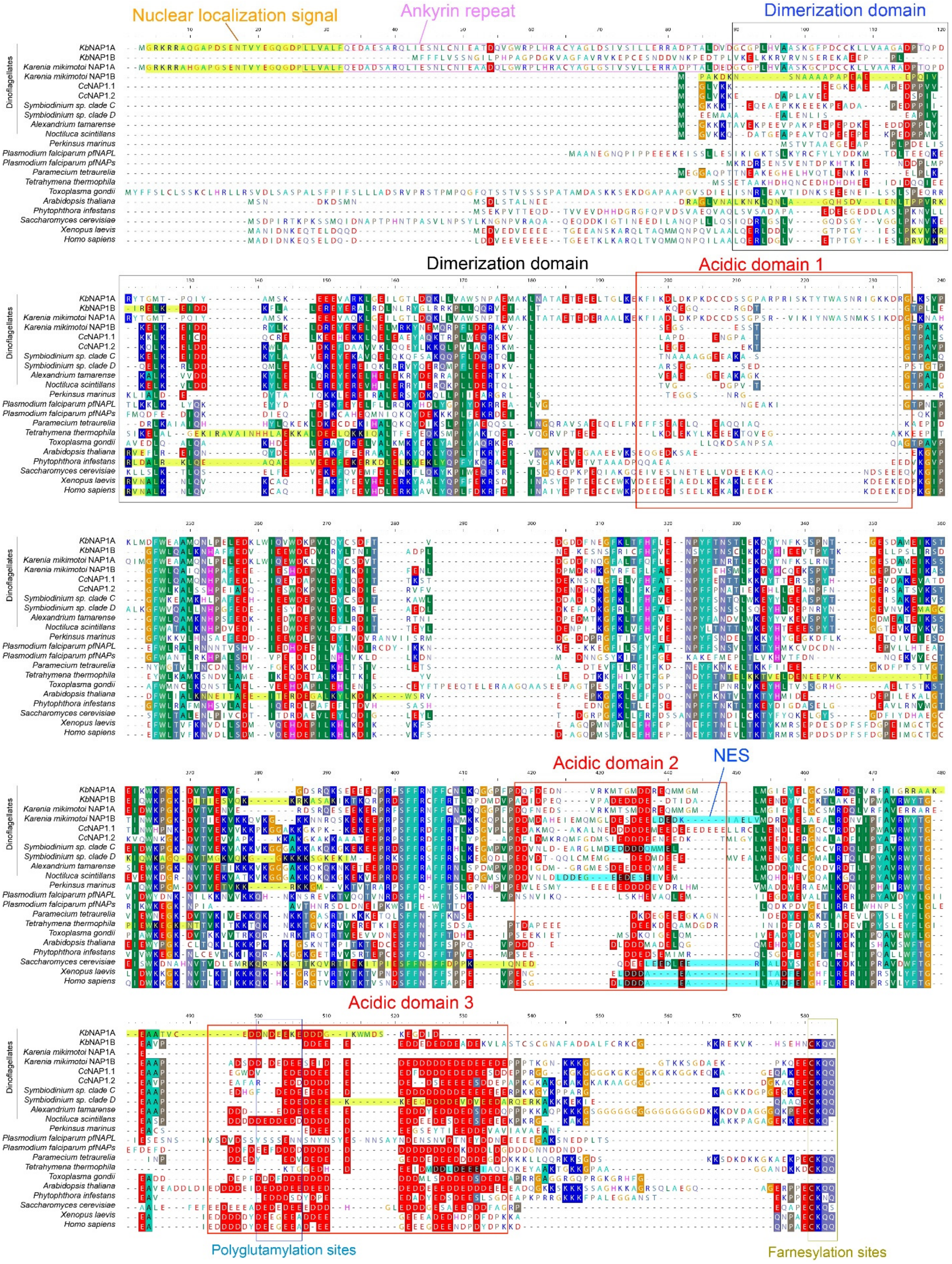

Figure S1. Multiple sequence alignment of dinoflagellate and selected eukaryotic NAP1 homologs.

Alignment of predicted polypeptide sequence of dinoflagellate and selected eukaryotic NAP1 homologs. Predicted nuclear localization signal (NLS) and nuclear export signals (NES) were also marked. KbNAP1Ap has longer putative dimerization domain (labelled) than the rest of dinoflagellates and non-dinoflagellates NAP1 homologs. All dinoflagellates NAP1 homologs, except KbNAP1Ap, have longer acidic domain 2 than other eukaryote homologs. Putative farnesylation sites ("CKQQ" motif) were identified on the C-terminal of NAP1 homologs. Acidic domains and polyglutamate tracks are highlighted based on previous studies (Miller and Heald 2015; Park and Luger 2006a; Park and Luger 2006b). Asterisk (*) indicates the overlapping of the NLS with the conserved polyglutamylation sites at the C-terminal of KbNAP1Ap. The residues are colored by Bioedit software with the default "Lesk" scheme, based on their chemical properties, with small non-polar residues (G, A, S, T) colored in orange, hydrophobic residues (C, V, I, L, P, F, Y, M, W) in green, polar residues (N, Q, H) in magenta, positively charged residues (K, R) colored in blue, negatively charged residues (D, E) in red.

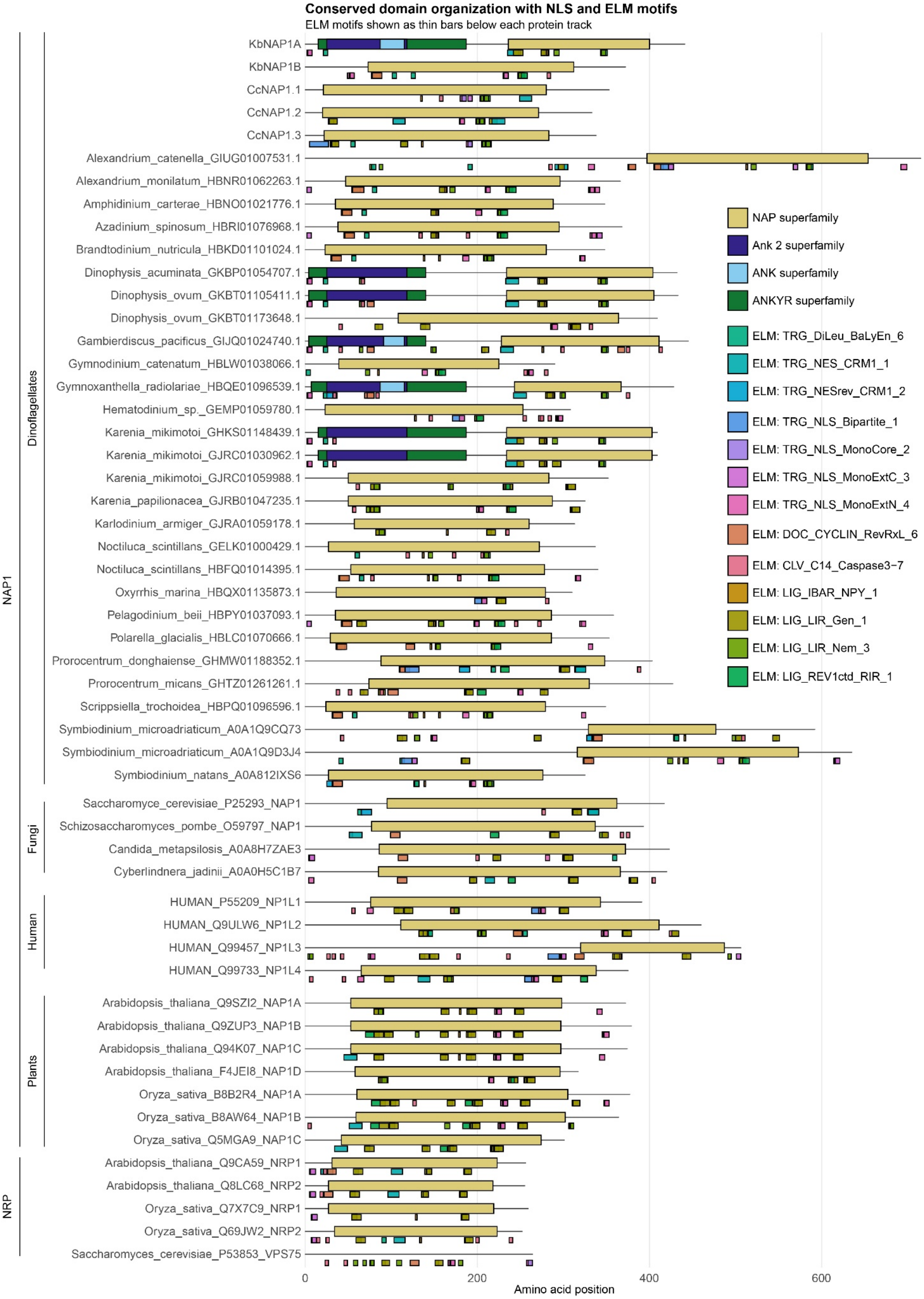

Conserved domain organization with NLS and ELM motifs
ELM motifs shown as thin bars below each protein track
NAP1
NRP
Dinoflagellates
Fungi
Human
Plants
KbNAP1A
KbNAP1B
CcNAP1.1
CcNAP1.2
CcNAP1.3
Alexandrium_catenella_GIUG01007531.1
Alexandrium_monilatum_HBNR01062263.1
Amphidinium_carterae_HBNO01021776.1
Azadinium_spinosum_HBRI01076968.1
Brandtodinium_nutricula_HBKD01101024.1
Dinophysis_acuminata_GKBP01054707.1
Dinophysis_ovum_GKBT01105411.1
Dinophysis_ovum_GKBT01173648.1
Gambierdiscus_pacificus_GIJQ01024740.1
Gymnodinium_catenatum_HBLW01038066.1
Gymnoxanthella_radiolariae_HBQE01096539.1
Hematodinium_sp._GEMP01059780.1
Karenia_mikimotoi_GHKS01148439.1
Karenia_mikimotoi_GJRC01030962.1
Karenia_mikimotoi_GJRC01059988.1
Karenia_papilionacea_GJRB01047235.1
Karlodinium_armiger_GJRA01059178.1
Noctiluca_scintillans_GELK01000429.1
Noctiluca_scintillans_HBFQ01014395.1
Oxyrrhis_marina_HBQX01135873.1
Pelagodinium_beii_HBPY01037093.1
Polarella_glacialis_HBLC01070666.1
Prorocentrum_donghaiense_GHMW01188352.1
Prorocentrum_micans_GHTZ01261261.1
Scrippsiella_trochoidea_HBPQ01096596.1
Symbiodinium_microadriaticum_A0A1Q9CQ73
Symbiodinium_microadriaticum_A0A1Q9D3J4
Symbiodinium_natans_A0A812IXS6
Saccharomyce_cerevisiae_P25293_NAP1
Schizosaccharomyces_pombe_O59797_NAP1
Candida_metapsilosis_A0A8H7ZAE3
Cyberlindnera_jadinii_A0A0H5C1B7
HUMAN_P55209_NP1L1
HUMAN_Q9ULW6_NP1L2
HUMAN_Q99457_NP1L3
HUMAN_Q99733_NP1L4
Arabidopsis_thaliana_Q9SZI2_NAP1A
Arabidopsis_thaliana_Q9ZUP3_NAP1B
Arabidopsis_thaliana_Q94K07_NAP1C
Arabidopsis_thaliana_F4JEI8_NAP1D
Oryza_sativa_B8B2R4_NAP1A
Oryza_sativa_B8AW64_NAP1B
Oryza_sativa_Q5MGA9_NAP1C
Arabidopsis_thaliana_Q9CA59_NRP1
Arabidopsis_thaliana_Q8LC68_NRP2
Oryza_sativa_Q7X7C9_NRP1
Oryza_sativa_Q69JW2_NRP2
Saccharomyces_cerevisiae_P53853_VPS75
0
200
400
600
Amino acid position
NAP superfamily
Ank 2 superfamily
ANK superfamily
ANKYR superfamily
ELM: TRG_DiLeu_BaLyEn_6
ELM: TRG_NES_CRM1_1
ELM: TRG_NESrev_CRM1_2
ELM: TRG_NLS_Bipartite_1
ELM: TRG_NLS_MonoCore_2
ELM: TRG_NLS_MonoExtC_3
ELM: TRG_NLS_MonoExtN_4
ELM: DOC_CYCLIN_RevRxL_6
ELM: CLV_C14_Caspase3-7
ELM: LIG_IBAR_NPY_1
ELM: LIG_LIR_Gen_1
ELM: LIG_LIR_Nem_3
ELM: LIG_REV1ctd_RIR_1

Figure S2. Conserved domain and Eukaryotic Linear Motif (ELM) organization of NAP1 and NAP1-related sequences across taxa.

Schematic map of conserved domain architecture (NCBI Conserved Domain Database) and predicted short linear motifs (ELM database; Kumar et al. 2024) plotted by amino acid position (x-axis) for the dinoflagellate NAP1 sequences analyzed in this study, together with comparator sequences from fungi, human, plants, and the plant-specific NAP1-Related Protein (NRP)/yeast-VPS75 clade. Colored rectangles denote conserved domain hits: the core NAP domain (NAP superfamily, Pfam PF00956, tan) and three related ankyrin-repeat domain calls (Ank_2 superfamily, dark blue; ANK superfamily, light blue; ANKYR superfamily, dark green), the latter three corresponding to the N-terminal ankyrin-repeat extension unique to KbNAP1A and select other dinoflagellate and plant NRP homologs. Small colored boxes beneath each domain track mark the positions of ELM-predicted motifs, restricted here to the motif classes discussed in the main text (Sections 2.1 and 3.2):

- TRG_NLS_MonoExtN_4 – N-extended monopartite classical NLS, the dominant nuclear import signal in dinoflagellate NAP1
- TRG_NLS_MonoExtC_3 – C-extended monopartite classical NLS
- TRG_NLS_MonoCore_2 – classical monopartite NLS, strong core variant
- TRG_NLS_Bipartite_1 – bipartite classical NLS
- TRG_NES_CRM1_1 – canonical leucine-rich nuclear export signal (NES) binding the CRM1 exportin
- TRG_NESrev_CRM1_2 – reverse-orientation NES binding the CRM1 groove in the minus direction
- DOC_CYCLIN_RevRxL_6 – reverse-orientation RxL-like docking motif for the Cyclin A2 hydrophobic patch
- CLV_C14_Caspase3-7 – Caspase-3/Caspase-7 proteolytic cleavage site
- LIG_IBAR_NPY_1 – NPY motif binding I-BAR domains, implicated in membrane curvature sensing and vesicular trafficking
- LIG_REV1ctd_RIR_1 – Rev1-Interacting Region (RIR) motif binding the C-terminal domain of the translesion DNA synthesis scaffold Rev1

Three independent *Karenia mikimotoi* entries are shown (GHKS01148439.1, GJRC01030962.1, and GJRC01059988.1), corresponding to distinct paralogous NAP1 loci rather than to redundant assemblies of a single gene, consistent with the NAP1A/NAP1B and NLS/NES-deficient paralog structure described in Section 2.1.

A

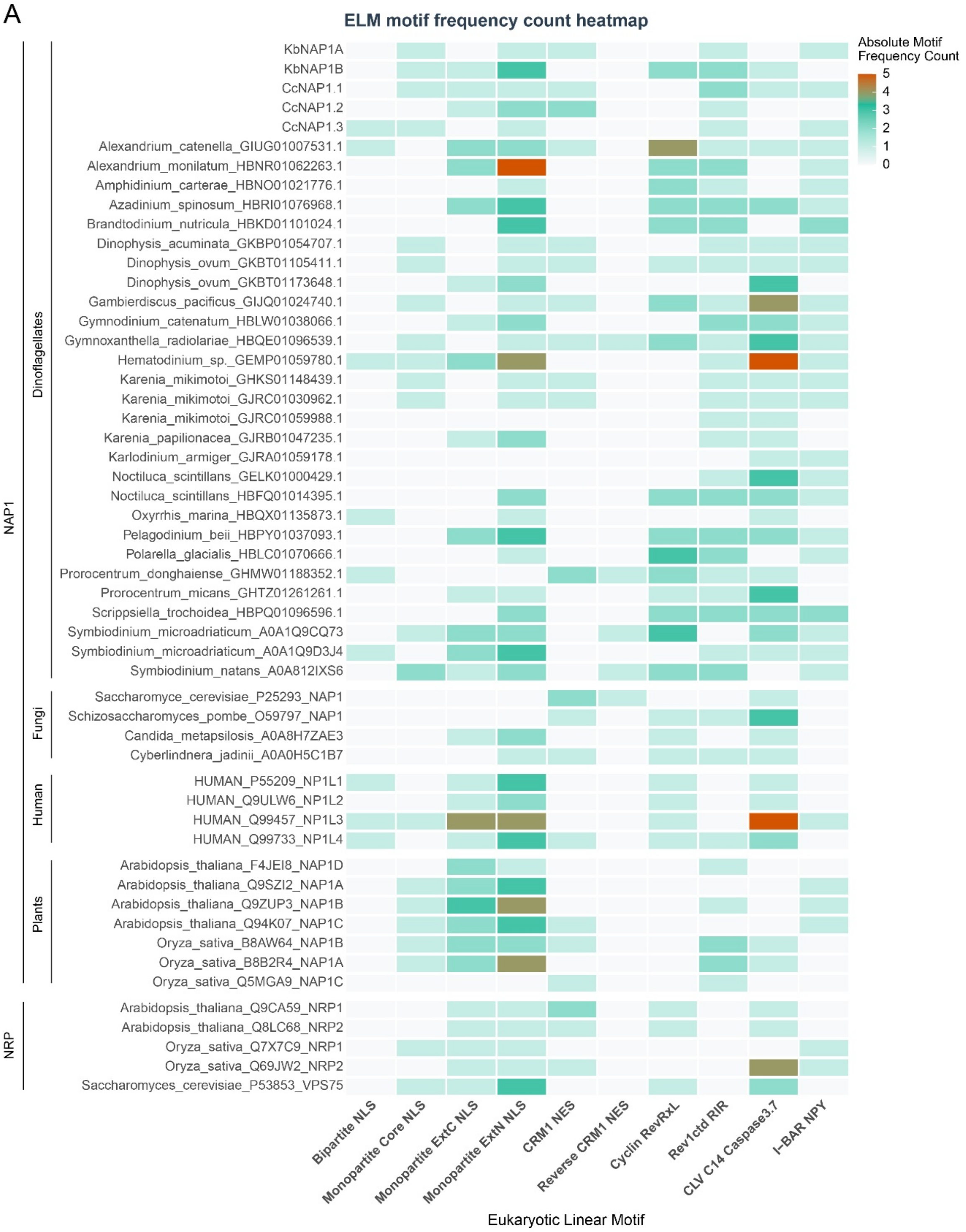

B

Motif Class	Dinoflagellate NAP1 (n=33)	Human NAP1 (n=4)	Plant NAP1 (n=7)	Yeast / Fungi NAP1 (n=4)
Monopartite ExtN NLS	87.9%	100.0%	85.7%	50.0%
Monopartite ExtC NLS	45.5%	100.0%	85.7%	25.0%
Monopartite Core NLS	39.4%	25.0%	71.4%	0.0%
Bipartite NLS	18.2%	75.0%	0.0%	0.0%
Overall NLS Presence	90.9%	100.0%	85.7%	50.0%
CRM1 NES	33.3%	25.0%	42.9%	75.0%
Reverse CRM1 NES	12.1%	0.0%	0.0%	25.0%
Overall NES Presence	39.4%	25.0%	42.9%	75.0%
Cyclin RevRxL	51.5%	100.0%	0.0%	75.0%
Rev1ctd RIR	87.9%	25.0%	71.4%	50.0%
Caspase 3/7 Cleavage	75.8%	100.0%	28.6%	100.0%
I-BAR NPY	75.8%	25.0%	42.9%	0.0%

Figure S3. Profiling of Eukaryotic Linear Motifs (ELMs) reveals an expanded non-canonical regulatory repertoire and atypical nucleocytoplasmic shuttling architectures in dinoflagellate NAP1.

(**A**) Heatmap depicting the absolute frequency counts of selected ELM classes across 53 representative NAP1 and NAP1-Related Protein (NRP) sequences from dinoflagellates, yeast/fungi, humans, and plants. (**B**) Summary table detailing the overall presence rates (percentage of sequences within a taxonomic group possessing at least one motif instance) for the analyzed ELM classes among canonical NAP1 orthologs. Dinoflagellate NAP1 (n=33) exhibits a robust overall NLS retention (90.9%) dominated by the N-extended monopartite variant (87.9%), alongside an intermediate overall NES presence (39.4%). Compared to human, plant, and yeast models, dinoflagellate NAP1 shows extraordinary, lineage-specific enrichment for several non-canonical interaction hubs, including the translesion DNA synthesis scaffold motif (Rev1ctd RIR; 87.9%), the membrane curvature and actin-dynamics tether (I-BAR NPY; 75.8%), and reverse-orientation Cyclin A2 docking sites (Cyclin RevRxL; 51.5%).

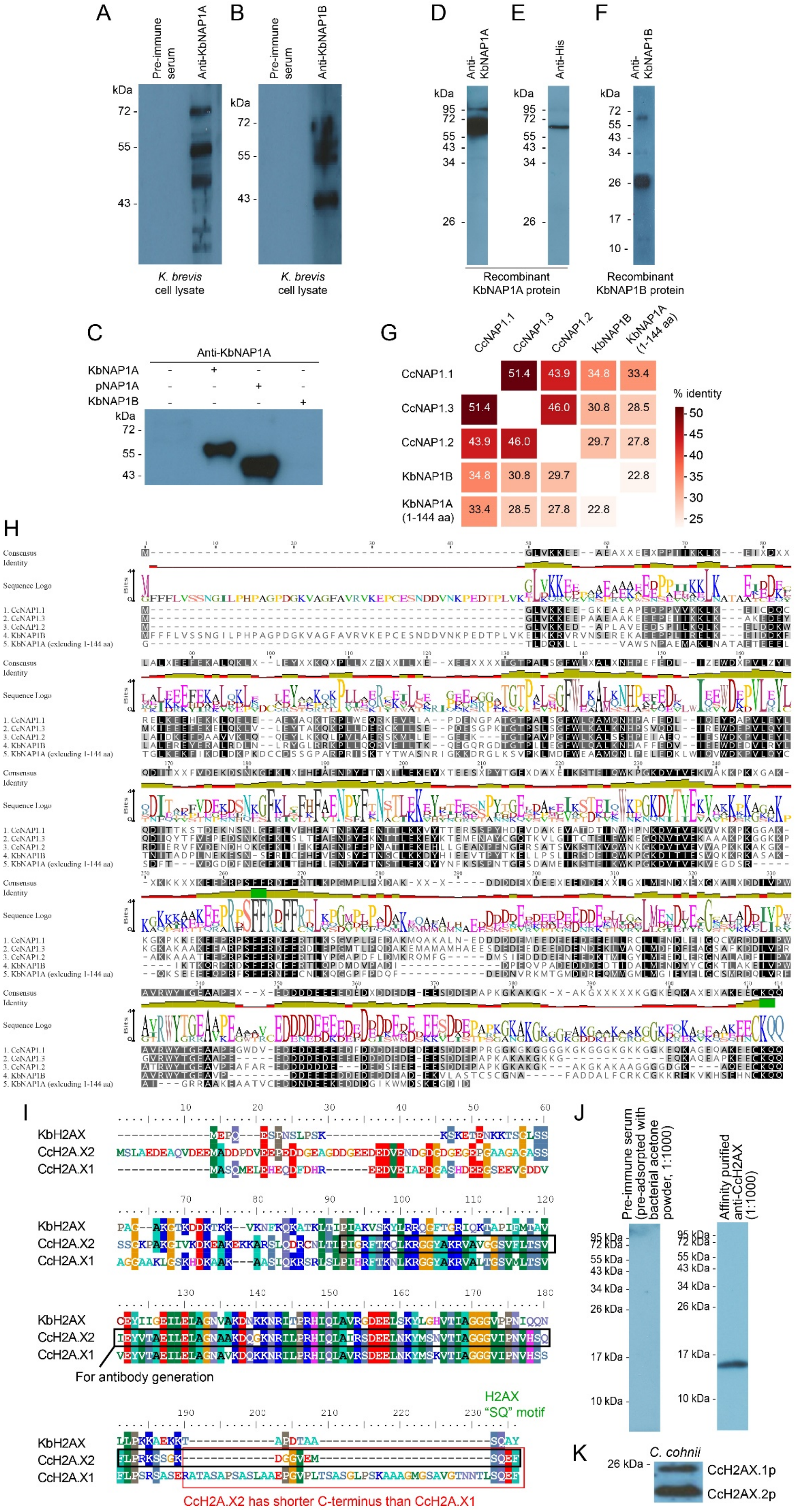

A
Pre-immune serum
Anti-KbNAP1A
kDa
72
55
43
K. brevis cell lysate
B
Pre-immune serum
Anti-KbNAP1B
kDa
72
55
43
K. brevis cell lysate
D
Anti-KbNAP1A
kDa
95
72
55
43
34
26
E
Anti-His
kDa
95
72
55
43
34
26
Recombinant KbNAP1A protein
F
Anti-KbNAP1B
kDa
72
55
43
34
26
17
10
Recombinant KbNAP1B protein
C
Anti-KbNAP1A
KbNAP1A - + - -
pNAP1A - - + -
KbNAP1B - - - +
kDa
72
55
43
G
CcNAP1.1
CcNAP1.3
CcNAP1.2
KbNAP1B
KbNAP1A (1-144 aa)
CcNAP1.1 51.4 43.9 34.8 33.4
CcNAP1.3 51.4 46.0 30.8 28.5
CcNAP1.2 43.9 46.0 29.7 27.8
KbNAP1B 34.8 30.8 29.7 22.8
KbNAP1A (1-144 aa) 33.4 28.5 27.8 22.8
% identity
50
45
40
35
30
25
H
Consensus
Identity
Sequence Logo
1. CcNAP1.1
2. CcNAP1.3
3. CcNAP1.2
4. KbNAP1B
5. KbNAP1A (exlcuding 1-144 aa)
I
KbH2AX
CcH2A.X2
CcH2A.X1
For antibody generation
H2AX "SQ" motif
CcH2A.X2 has shorter C-terminus than CcH2A.X1
J
Pre-immune serum (pre-adsorpted with bacterial acetone powder, 1:1000)
Affinity purified anti-CcH2AX (1:1000)
95 kDa
72 kDa
55 kDa
43 kDa
34 kDa
26 kDa
17 kDa
10 kDa
K
C. cohnii
26 kDa
CcH2AX.1p
CcH2AX.2p

Figure S4. Validation of anti-KbNAP1A, anti-KbNAP1B, and anti-CcH2AX.2 antibodies and characterization of recombinant NAP1 proteins.

(**A**) Immunoblots of *K. brevis* total cell lysates probed with anti-KbNAP1A antibody or the corresponding pre-immune serum, showing specific immunoreactive bands at ~72 kDa and ~55 kDa with the specific antibody and absent in pre-immune controls. (**B**) Immunoblots of *K. brevis* total cell lysates probed with anti-KbNAP1B antibody or the corresponding pre-immune serum, showing specific immunoreactive bands at ~50 kDa and ~43 kDa. (**C**) Antibody specificity test: anti-KbNAP1A did not cross-react with KbNAP1Bp. Lane 1, bacterial lysate with no protein expression; Lane 2, recombinant KbNAP1Ap expression; Lane 3, pNAP1Ap (ankyrin repeat-deleted KbNAP1A) expression; Lane 4, recombinant KbNAP1Bp expression. (**D**) Immunoblot of purified recombinant KbNAP1Ap probed with anti-KbNAP1A, detecting the 61 kDa monomer and a higher-molecular-weight dimer. (**E**) Immunoblot of purified recombinant KbNAP1Ap probed with anti-His; the anti-His antibody failed to recognize the KbNAP1A dimer, consistent with occlusion of the N-terminal His-tag within the dimerization interface. Apparent molecular weights in *K. brevis* cell lysates differ from bacterially expressed proteins due to PTMs absent in the prokaryotic expression system. (**F**) Immunoblot of purified recombinant KbNAP1Bp probed with anti-KbNAP1B. Additional antibody pre-depletion specificity control is shown in Figure S7. (**G**) Identity of the antibody antigens to the *C. cohnii* NAP1 isoforms. Pairwise amino-acid between the immunogen regions, KbNAP1A residues 145–441 (ankyrin repeat excluded) and full-length KbNAP1B, and the homologous, gap-trimmed regions of CcNAP1.1, CcNAP1.2 and CcNAP1.3, computed with SIAS over the alignment of (**H**). KbNAP1A antigen vs CcNAP1.1/1.2/1.3: 27.8-33.4% identity; KbNAP1B antigen vs CcNAP1.1/1.2/1.3: 29.7-34.8% identity. (**I**) Multiple sequence alignment of H2AX variants from *C. cohnii* (CcH2AX.1, 214 aa, pI 5.75; CcH2AX.2, 199 aa, pI 4.4) and *K. brevis* (KbH2AX, 17.9 kDa predicted MW, >40% homology to CcH2AX.2). CcH2AX.2 has a longer N-terminus but shorter C-terminus than CcH2AX.1; the two homologs are predicted to co-migrate on SDS-PAGE. Residues 93–200 (boxed), encompassing the shared histone fold domain, were used as antigen for anti-CcH2AX.2 generation. The canonical H2AX "SQ" phosphorylation motif is indicated. (**J**) Affinity-purified anti-CcH2AX.2 antibody detected a ~14 kDa bacterially expressed truncated CcH2AX.2p (antigen) by immunoblot. (**K**) Both CcH2AX.1p and CcH2AX.2p were detected in *C. cohnii* total cell lysate at approximately 26 kDa. Approximately 50 μg total protein per lane was loaded in (**J**) and (**K**).

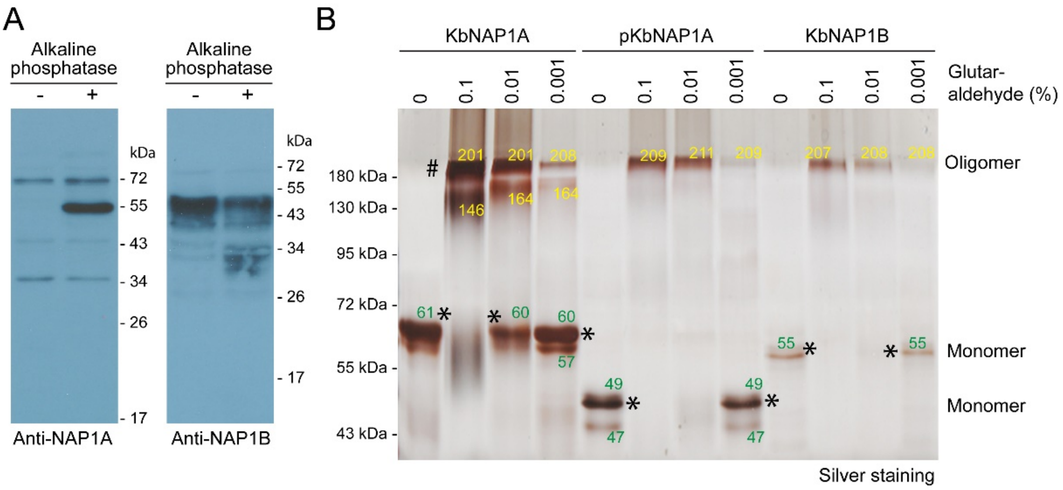

Figure S5. Post-translational modification profiling and oligomerization analysis of dinoflagellate NAP1 proteins.

(**A**) Phosphorylation state analysis by alkaline phosphatase treatment. Total *K. brevis* cell lysates were incubated with active (+) or heat-inactivated (−) calf intestinal alkaline phosphatase (CIP; NEB) at 37°C for 30 min, resolved by SDS-PAGE, and immunoblotted with anti-KbNAP1A (left panel) and anti-KbNAP1B (right panel). On this basis, the ~72 kDa band observed for KbNAP1A is consistent with the fully phosphorylated and mono-ubiquitinated form (1Ub + 12P; theoretical +8.5 kDa ubiquitination + 12 kDa phosphorylation applied to the 49.8

kDa predicted MW, calculated mass 70.3 kDa); the ~55 kDa band is consistent with the unphosphorylated, non-ubiquitinated form bearing five phosphorylation events (5P; calculated mass 54.8 kDa). For KbNAP1B: the ~50 kDa band corresponds to the mono-ubiquitinated, unphosphorylated form (1Ub; calculated mass 51.7 kDa) and the ~43 kDa band to the unphosphorylated form (0P). Dephosphorylation (active CIP) collapsed the upper phosphorylated bands, confirming phosphorylation-dependent mobility shifts. See Table S3 for full PTM annotations. (**B**) Concentration-dependent oligomerization of recombinant NAP1 proteins assessed by glutaraldehyde cross-linking. Recombinant KbNAP1Ap (full-length, ~61 kDa), pKbNAP1Ap (ankyrin repeat-deleted, ~49 kDa), and KbNAP1Bp (~55 kDa) were cross-linked with 0 (uncrosslinked control), 0.001%, 0.01%, or 0.1% (w/v) glutaraldehyde in PBS pH 7.4 for 30 min at 22°C, resolved by SDS-PAGE, and visualized by silver staining. Asterisks (*) indicate monomer bands; hash (#) indicates putative oligomer bands (~150–200 kDa, consistent with dimeric to tetrameric species). KbNAP1A exhibited the least oligomerization propensity among all three forms; pKbNAP1A (ankyrin repeat-deleted) formed higher-order oligomers more readily than full-length KbNAP1A, demonstrating that the ankyrin repeat domain sterically suppresses oligomerization. KbNAP1B showed the strongest affinity for higher-order oligomer formation. Elevated glutaraldehyde concentrations (0.1%) reduced detectable oligomers, likely due to structural denaturation disrupting protein–protein contact surfaces.

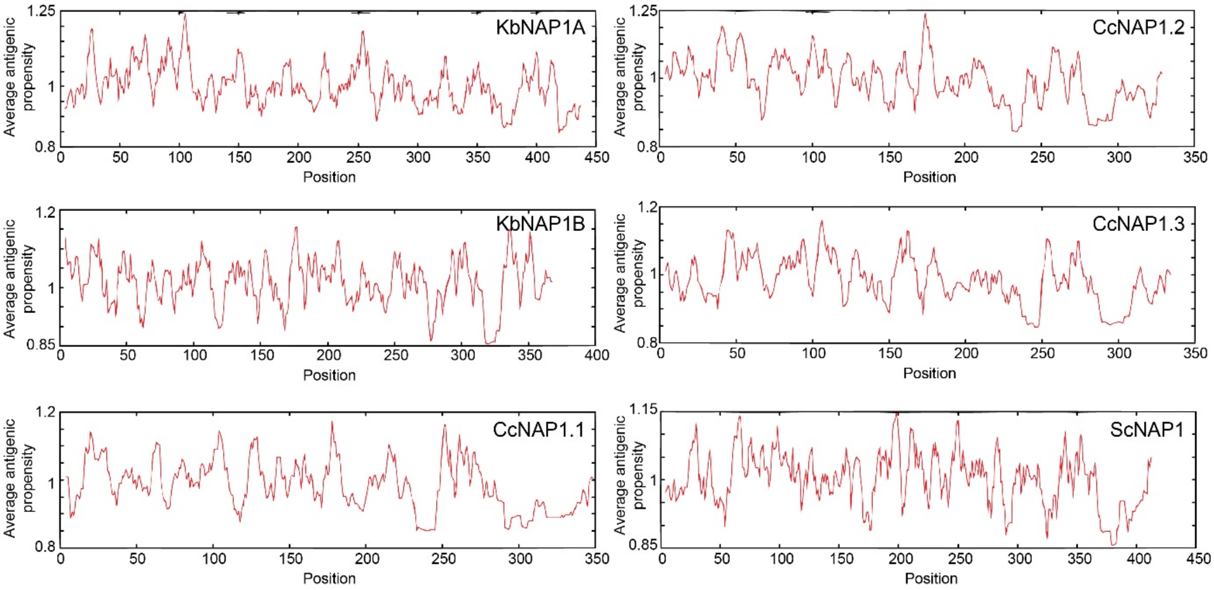

Figure S6. EMBOSS antigenicity profiles of dinoflagellate NAP1 homologs.

Average antigenic propensity along the polypeptide sequences of six NAP1 homologs, KbNAP1A (441 aa), KbNAP1B (372 aa), CcNAP1.1 (353 aa), CcNAP1.2 (333 aa), CcNAP1.3 (338 aa), and ScNAP1 (417 aa), calculated using the EMBOSS Antigenic program with the Kolaskar–Tongaonkar semi-empirical method (Kolaskar and Tongaonkar 1990). Red lines indicate the running antigenic propensity score (y-axis range 0.80–1.25). Predicted antigenic determinants (peaks above threshold 1) are distributed throughout all homologs, confirming their suitability for antibody generation. Antigenicity profiles were used to guide selection of immunogenic peptide regions. Both KbNAP1A and KbNAP1B display relatively uniform antigenicity across the NAP domain region, consistent with the experimentally confirmed antibody reactivity (Figure S4). Profiles for CcNAP1.1, CcNAP1.2, and CcNAP1.3 show comparable antigenic patterns, consistent with the cross-reactivity of *K. brevis* antibodies to *C. cohnii* isoforms.

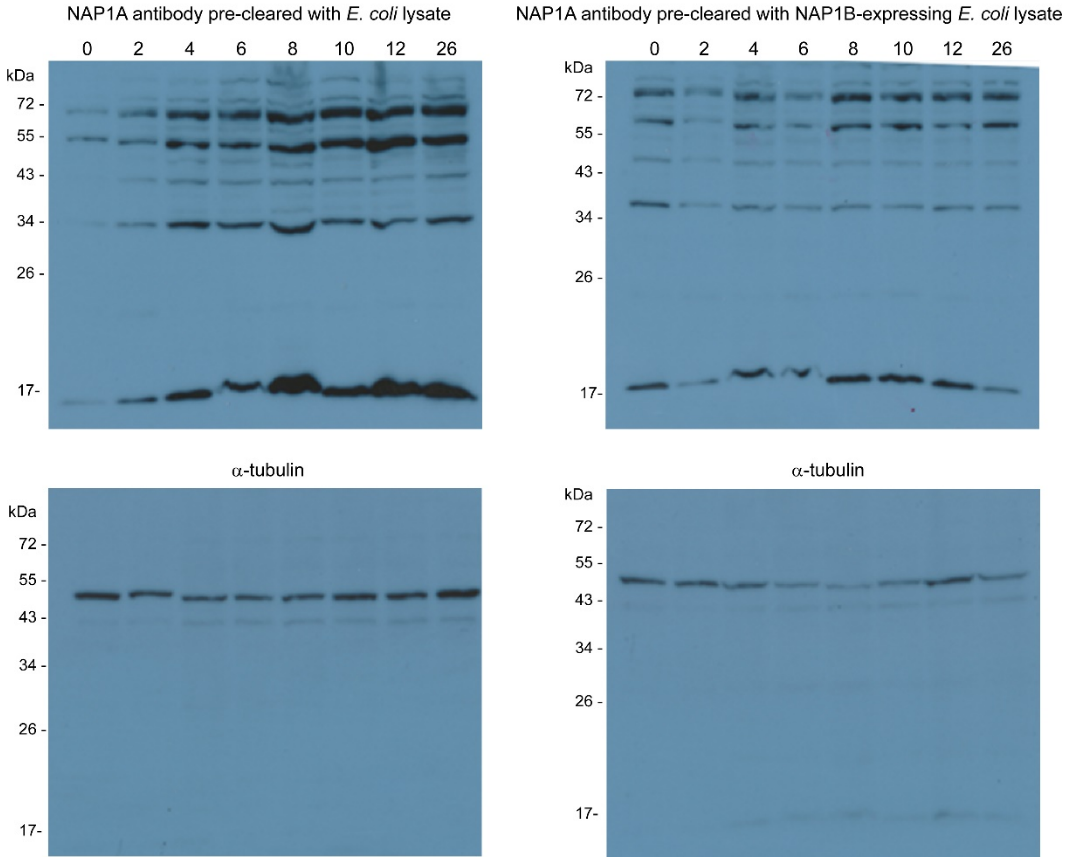

Figure S7. Confirmation of antibody specificity by heterologous recombinant protein pre-depletion.

Immunoblot of *C. cohnii* total cell lysates (T = 0, 2, 4, 6, 8, 10, 12, 26 hr after synchronization) probed with anti-KbNAP1A antibody pre-cleared against E. coli lysate (left panels) or anti-KbNAP1A antibody pre-depleted against full-length recombinant KbNAP1Bp-expressing *E. coli* lysate (right panels). Upper panels: anti-KbNAP1A immunoblot; lower panels: anti-α-tubulin loading control. The immunoblot patterns are essentially identical between the two pre-treatment conditions, confirming the absence of antigenic overlap between KbNAP1Ap and KbNAP1Bp and validating the specificity of the anti-KbNAP1A antibody for *C. cohnii* NAP1 isoforms.

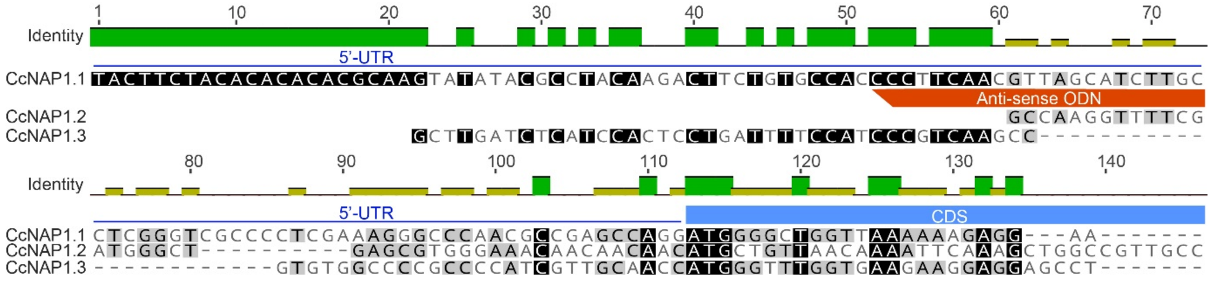

Figure S8. The CcNAP1.1 antisense target diverges from the CcNAP1.2 and CcNAP1.3 5′-UTRs.

Alignment of the 5′-untranslated regions of CcNAP1.1 (112 bp), CcNAP1.2 and CcNAP1.3. The 21-nt antisense-ODN target (positions 53–73 of the CcNAP1.1 5′-UTR; orange bar) aligns to the CcNAP1.2 and CcNAP1.3 5′-UTRs with only 23.8% and 38.1% identity, confirming that the knockdown ODN is isoform-selective for CcNAP1.1.

Table S1. Core histone orthologues identified in *Crypthecodinium cohnii*.

GenBank accession numbers, amino acid lengths, and predicted molecular weights (kDa) of *C. cohnii* core histone variants and histone-like proteins identified by transcriptome analysis, including H2AZ, H2AX.1, H2AX.2, H2B.1–H2B.3, H3.1–H3.6, and H4.1–H4.2. Calculated molecular weight ranges for histone sub-complexes (H2AX/H2B dimer, H3/H4 dimer, H3/H4 tetramer, and full histone octamer) are provided based on the sequence data; ranges reflect the diversity of histone isoforms.

**Histone**	**Sequence accession no.**	**No. of residues (amino acids)**	**MW (kDa)**
H2AZ	MN889533	269	29.2
H2AX.1	MN889534	214	22.6
H2AX.2	MN889535	199	21.1
H2B.1	MN889536	161	17.7
H2B.2	MN889537	137	14.7
H2B.3	MN889538	122	13.7
H3.1	MT956610	189	20.5
H3.2	MT956611	187	20.5
H3.3	MT956612	175	16.1
H3.4	MT956613	158	17.5
H3.5	MT956614	171	18.3
H3.6	MT956615	165	18.2
H4.1	MT956616	228	23.3
H4.2	MT956617	114	12.5
H2AX/H2B dimer			34.8 – 46.9
H3/H4 dimer			28.6 – 43.8
H3/H4 tetramer			57.2 – 87.6
Histone octamer (2 x H2AX/H2B dimer + H3/H4 tetramer)			126.8 – 181.4

Footnote: H3/H4 dimer minimum reflects the smallest H3 (H3.3, 16.1 kDa) paired with the smallest H4 (H4.2, 12.5 kDa).

Table S2. NAP1 protein properties.

Theoretical monomer and homodimer molecular weights (kDa), isoelectric points (pI), predicted numbers of phosphorylation and ubiquitination sites (MusiteDeep, threshold 0.5), apparent molecular weights of immunoreactive bands, and inferred PTM states for *K. brevis* NAP1A/NAP1B and *C. cohnii* NAP1.1–1.3. Observed apparent molecular weights and inferred PTM combinations for immunoreactive bands are presented separately in Table S4.

Species	Protein	Predicted pI	Monomer (kDa)	Dimer (kDa)	Predicted P sites (+1 kDa)	Predicted Ub sites (+8.5 kDa)
K. brevis	NAP1A	4.67	49.8	99.6	12	4
K. brevis	NAP1B	4.83	43.2	86.4	5	5
C. cohnii	NAP1.1	4.48	40.1	80.2	4	1
C. cohnii	NAP1.2	4.64	37.5	75.0	7	4
C. cohnii	NAP1.3	4.43	38.6	77.2	9	3

Table S3. Western band assignments and inferred combinatorial PTM states for dinoflagellate NAP1 immunoreactive species.

Each phosphorylation contributes ≈+1 kDa and each ubiquitination ≈+8.5 kDa to apparent molecular weight. Apparent molecular weights consistently exceed predicted values owing to the combined effects of anomalous SDS-PAGE migration characteristic of highly acidic proteins (pI 4.4–4.8; (Tiwari et al. 2019; Guan et al. 2015)), which accounts for the baseline offset of the unmodified form, and PTMs, which account for the shifts between bands of the same homolog (directly supported by the alkaline-phosphatase band collapse in Figure S4A). The ~69 kDa anti-KbNAP1A band in *C. cohnii* is consistent with heavily modified CcNAP1.2 (≈6P + 3Ub) or CcNAP1.3 (≈5P + 3Ub) within their predicted site budgets; the specific isoform and modification pattern remain to be confirmed experimentally, and combinations requiring three ubiquitin moieties are considered tentative. *C. cohnii* band assignments are based on anti-KbNAP1A/anti-KbNAP1B cross-reactivity and should be regarded as provisional. Related to Figure S4A.

Species	Antibody	Band (kDa)	Protein	Assigned PTMs	Calc. mass (kDa)	Fit
K. brevis	anti-NAP1A	72	NAP1A	1Ub + 12P	70.3	✓
		68	NAP1A	1Ub + 10P *(or 2Ub + 2P)*	68.3	✓
		55	NAP1A	5P	54.8	✓
K. brevis	anti-NAP1B	50	NAP1B	1Ub	51.7	✓
		43	NAP1B	0 PTM	43.2	✓
C. cohnii	anti-NAP1A	69	NAP1.2	3Ub + 6P	69.0	✓
			NAP1.3	3Ub + 5P	69.1	✓
			NAP1.1	—	max ~52.6	✗
		54	NAP1.2	2Ub	54.5	✓
			NAP1.3	2Ub *(or 1Ub + 7P = 54.1)*	55.6	✓
			NAP1.1	—	max ~52.6	✗
C. cohnii	anti-NAP1B	52	NAP1.1	1Ub + 4P	52.6	✓
			NAP1.2	1Ub + 6P	52.0	✓
			NAP1.3	1Ub + 5P	52.1	✓
		48	NAP1.1	1Ub	48.6	✓
			NAP1.2	1Ub + 2P	48.0	✓
			NAP1.3	1Ub + 1P *(or 9P = 47.6)*	48.1	✓
		38	NAP1.1	0 PTM	40.1	≈

			NAP1.2	0 PTM	37.5	✓
			NAP1.3	0 PTM	38.6	✓

Table S4: Exact amino acid sequence motifs of predicted post-translational modification (PTM) sites in dinoflagellate and some selected NAP1 homologs. Post-translational modifications, including phosphorylation, ubiquitination, acetylation, methylation, and SUMOylation, were predicted using MusiteDeep. Only significant predictions with a confidence score of ≥ 0.5 are listed. For each predicted PTM, the local sequence context is provided as an 11-amino-acid window (±5 residues flanking the modification site). The specific central residue undergoing the predicted modification is highlighted in red. Dashes (-) indicate positions where the sequence window extends beyond the N- or C-terminus of the protein.

Protein ID	Position	Residue	Predicted PTM	Score	Sequence Motif
CcNAP1.1	5	K	SUMOylation	0.947	-MGLVKKEEGK
CcNAP1.1	5	K	Methyllysine	0.628	-MGLVKKEEGK
CcNAP1.1	5	K	N6-acetyllysine	0.546	-MGLVKKEEGK
CcNAP1.1	6	K	SUMOylation	0.920	MGLVKKEEGKE
CcNAP1.1	35	K	SUMOylation	0.533	QCRELKEEHEK
CcNAP1.1	139	N	N-linked_glycosylation	0.900	NPYFENTTLKK
CcNAP1.1	148	T	O-linked_glycosylation	0.633	KKVYTTERSSP
CcNAP1.1	151	S	Phosphoserine	0.651	YTTERSSPYHD
CcNAP1.1	152	S	Phosphoserine	0.865	TTERSSPYHDE
CcNAP1.1	174	K	Ubiquitination	0.534	NWHPNKDVTVE
CcNAP1.1	180	K	N6-acetyllysine	0.634	DVTVEKVVKKP
CcNAP1.1	204	S	Phosphoserine	0.527	EEPRPSFFRDF
CcNAP1.1	224	K	N6-acetyllysine	0.575	LPEDAKMQAKA
CcNAP1.1	310	S	Phosphoserine	0.883	DEDEESDDEPP
CcNAP1.1	310	S	O-linked_glycosylation	0.656	DEDEESDDEPP
CcNAP1.1	316	R	Methylarginine	0.674	DDEPPRGGKGK
CcNAP1.1	321	K	N6-acetyllysine	0.559	RGGKGKGGGGK
CcNAP1.1	326	K	N6-acetyllysine	0.841	KGGGGKGKGGG
CcNAP1.1	328	K	N6-acetyllysine	0.845	GGGKGKGGGKG
CcNAP1.1	332	K	N6-acetyllysine	0.851	GKGGGKGKKGG
CcNAP1.1	334	K	N6-acetyllysine	0.834	GGGKGKKGGKE

Protein ID	Position	Residue	Predicted PTM	Score	Sequence Motif
CcNAP1.1	335	K	N6-acetyllysine	0.762	GGKGK**K**GGKEQ
CcNAP1.1	338	K	N6-acetyllysine	0.729	GKKGG**K**EQKAG
CcNAP1.1	347	K	SUMOylation	0.619	AGEQA**K**EECKQ
CcNAP1.1	347	K	N6-acetyllysine	0.501	AGEQA**K**EECKQ
CcNAP1.2	5	K	SUMOylation	0.940	-MGLV**K**KEDAP
CcNAP1.2	5	K	Methyllysine	0.502	-MGLV**K**KEDAP
CcNAP1.2	6	K	Ubiquitination	0.581	MGLVK**K**EDAPL
CcNAP1.2	6	K	SUMOylation	0.611	MGLVK**K**EDAPL
CcNAP1.2	17	S	Phosphoserine	0.881	AVEED**S**PILKQ
CcNAP1.2	29	K	N6-acetyllysine	0.524	KELDD**K**WLAID
CcNAP1.2	35	K	Ubiquitination	0.613	WLAID**K**EFDAA
CcNAP1.2	69	K	Ubiquitination	0.550	LEGEE**K**TGTPA
CcNAP1.2	72	T	Phosphothreonine	0.545	EEKTG**T**PAVPG
CcNAP1.2	81	K	Ubiquitination	0.566	PGFWL**K**ALSSH
CcNAP1.2	135	N	N-linked_glycosylation	0.908	NPFFP**N**ATIEK
CcNAP1.2	140	K	Ubiquitination	0.609	NATIE**K**EHLLG
CcNAP1.2	173	T	Phosphothreonine	0.631	KGKDV**T**VEVVA
CcNAP1.2	199	S	Phosphoserine	0.529	EEPRP**S**FFRDF
CcNAP1.2	227	S	Phosphoserine	0.914	MFGDM**S**IEDDD
CcNAP1.2	291	S	Phosphoserine	0.915	DEDED**S**EEEEE
CcNAP1.2	291	S	O-linked_glycosylation	0.511	DEDED**S**EEEEE
CcNAP1.2	297	S	Phosphoserine	0.838	EEEEE**S**DDEPA
CcNAP1.2	308	K	N6-acetyllysine	0.604	PKGKA**K**GKAKG
CcNAP1.2	310	K	N6-acetyllysine	0.710	GKAKG**K**AKGKA
CcNAP1.2	312	K	N6-acetyllysine	0.752	AKGKA**K**GKAKA
CcNAP1.2	314	K	N6-acetyllysine	0.752	GKAKG**K**AKAAG
CcNAP1.2	316	K	N6-acetyllysine	0.765	AKGKA**K**AAGGG

Protein ID	Position	Residue	Predicted PTM	Score	Sequence Motif
CcNAP1.2	325	K	N6-acetyllysine	0.663	GGGDGKAQEEC
CcNAP1.3	5	K	SUMOylation	0.954	-MGLVKKEEPA
CcNAP1.3	5	K	N6-acetyllysine	0.590	-MGLVKKEEPA
CcNAP1.3	6	K	Ubiquitination	0.567	MGLVKKEEPAA
CcNAP1.3	6	K	SUMOylation	0.942	MGLVKKEEPAA
CcNAP1.3	6	K	N6-acetyllysine	0.523	MGLVKKEEPAA
CcNAP1.3	15	K	Ubiquitination	0.568	AAEAAKEEEPE
CcNAP1.3	15	K	SUMOylation	0.826	AAEAAKEEEPE
CcNAP1.3	34	K	SUMOylation	0.536	EDEYMKIEEEF
CcNAP1.3	66	S	Phosphoserine	0.715	RCKILSEPQES
CcNAP1.3	71	S	Phosphoserine	0.533	SEPQESGPKTG
CcNAP1.3	77	T	Phosphothreonine	0.652	GPKTGTPSLSG
CcNAP1.3	93	S	Phosphoserine	0.567	LKNHPSVQDLI
CcNAP1.3	139	K	Ubiquitination	0.570	ENPYFKNTTLE
CcNAP1.3	140	N	N-linked_glycosylation	0.909	NPYFKNTTLEK
CcNAP1.3	145	K	N6-acetyllysine	0.688	NTTLEKEYKTE
CcNAP1.3	176	N	N-linked_glycosylation	0.908	WKEGQNVTVEK
CcNAP1.3	200	K	SUMOylation	0.846	KAKAAKEEARP
CcNAP1.3	206	S	Phosphoserine	0.598	EEARPSFFRDF
CcNAP1.3	226	K	Ubiquitination	0.534	LPQDAKEMAMA
CcNAP1.3	237	S	Phosphoserine	0.920	MHAEESDEDEE
CcNAP1.3	252	K	Ubiquitination	0.516	DENDEKLVAQL
CcNAP1.3	280	Y	Phosphotyrosine	0.686	RGVRWYTGEAA
CcNAP1.3	281	T	Phosphothreonine	0.591	GVRWYTGEAAP
CcNAP1.3	308	S	Phosphoserine	0.881	EDEEESDDEPA
CcNAP1.3	308	S	O-linked_glycosylation	0.590	EDEEESDDEPA
CcNAP1.3	322	K	SUMOylation	0.700	KAKGKKGEQKA

Protein ID	Position	Residue	Predicted PTM	Score	Sequence Motif
CcNAP1.3	332	K	SUMOylation	0.715	AQEAPKEECKQ
HUMAN_NAP1L1	7	K	Ubiquitination	0.725	ADIDNKEQSEL
HUMAN_NAP1L1	10	S	Phosphoserine	0.864	DNKEQSELDQD
HUMAN_NAP1L1	27	T	Phosphothreonine	0.676	VEEEETGEETK
HUMAN_NAP1L1	62	T	Phosphothreonine	0.784	DGLVETPTGYI
HUMAN_NAP1L1	64	T	Phosphothreonine	0.683	LVETPTGYIES
HUMAN_NAP1L1	69	S	Phosphoserine	0.801	TGYIESLPRVV
HUMAN_NAP1L1	116	K	N6-acetyllysine	0.816	QPLFDKRFEII
HUMAN_NAP1L1	143	S	Phosphoserine	0.879	EEDEISEELKE
HUMAN_NAP1L1	157	K	SUMOylation	0.543	IEDEKKDEEKE
HUMAN_NAP1L1	237	S	Phosphoserine	0.842	TYRMRSEPDDS
HUMAN_NAP1L1	242	S	Phosphoserine	0.659	SEPDDSDPFSF
HUMAN_NAP1L1	246	S	Phosphoserine	0.520	DSDPFSFDGPE
HUMAN_NAP1L1	267	N	N-linked_glycosylation	0.880	WKKGKNVTLKT
HUMAN_NAP1L1	282	R	Methylarginine	0.779	QKHKGRGTVRT
HUMAN_NAP1L1	293	S	Phosphoserine	0.639	VTKTVSNDSFF
HUMAN_NAP1L1	294	N	N-linked_glycosylation	0.890	TKTVSNDSFFN
HUMAN_NAP1L1	309	S	Phosphoserine	0.631	PEVPESGDLDD
HUMAN_NAP1L1	309	S	O-linked_glycosylation	0.663	PEVPESGDLDD
HUMAN_NAP1L1	341	Y	Phosphotyrosine	0.635	PRSVLYFTGEA
HUMAN_NAP1L1	343	T	O-linked_glycosylation	0.568	SVLYFTGEAIE
HUMAN_NAP1L1	354	Y	Phosphotyrosine	0.631	DDDDDYDEEGE
HUMAN_NAP1L1	380	K	Ubiquitination	0.604	PDYDPKKDQNP
HUMAN_NAP1L1	381	K	Ubiquitination	0.613	DYDPKKDQNPA
KbNAP1A	4	K	Methyllysine	0.803	--MGRKRRAQG
KbNAP1A	13	S	Phosphoserine	0.660	QGAPDSENTVY
KbNAP1A	18	Y	Phosphotyrosine	0.844	SENTVYEGQGD

Protein ID	Position	Residue	Predicted PTM	Score	Sequence Motif
KbNAP1A	124	T	Phosphothreonine	0.639	RYTGMTPQIYA
KbNAP1A	163	K	Ubiquitination	0.503	PAEMAKLNATA
KbNAP1A	165	N	N-linked_glycosylation	0.908	EMAKLNATAET
KbNAP1A	167	T	O-linked_glycosylation	0.584	AKLNATAETEE
KbNAP1A	170	T	Phosphothreonine	0.604	NATAETEEELT
KbNAP1A	204	K	Methyllysine	0.523	RPRISKTYTWA
KbNAP1A	204	K	N6-acetyllysine	0.652	RPRISKTYTWA
KbNAP1A	285	N	N-linked_glycosylation	0.915	NPYFTNSTLEK
KbNAP1A	297	S	Phosphoserine	0.617	YYNFKSSPNTG
KbNAP1A	298	S	Phosphoserine	0.818	YNFKSSPNTGE
KbNAP1A	301	T	Phosphothreonine	0.643	KSSPNTGESDA
KbNAP1A	304	S	Phosphoserine	0.648	PNTGESDAMEI
KbNAP1A	326	K	N6-acetyllysine	0.525	DVTVEKVEGDS
KbNAP1A	331	S	Phosphoserine	0.530	KVEGDSRQKSE
KbNAP1A	334	K	Ubiquitination	0.628	GDSRQKSEEEE
KbNAP1A	335	S	Phosphoserine	0.850	DSRQKSEEEEQ
KbNAP1A	414	T	Phosphothreonine	0.615	AKEAATVCEDD
KbNAP1A	431	K	Ubiquitination	0.578	DDDGIKWMDSK
KbNAP1A	435	S	Phosphoserine	0.841	IKWMDSKEGDI
KbNAP1A	436	K	Ubiquitination	0.617	KWMDSKEGDID
KbNAP1B	30	K	SUMOylation	0.504	FAVRVKEPCES
KbNAP1B	35	S	Phosphoserine	0.786	KEPCESNDDVN
KbNAP1B	45	T	Phosphothreonine	0.755	NKPEDTPLVKE
KbNAP1B	49	K	N6-acetyllysine	0.667	DTPLVKELKKR
KbNAP1B	59	S	Phosphoserine	0.789	RVRVNSEREKA
KbNAP1B	63	K	Ubiquitination	0.506	NSEREKAEEPP
KbNAP1B	74	K	Ubiquitination	0.566	LIRELKEIDDK

Protein ID	Position	Residue	Predicted PTM	Score	Sequence Motif
KbNAP1B	79	K	Ubiquitination	0.626	KEIDDKFLALE
KbNAP1B	79	K	N6-acetyllysine	0.567	KEIDDKFLALE
KbNAP1B	103	K	Ubiquitination	0.694	YGLRRKPLLQQ
KbNAP1B	115	K	Ubiquitination	0.534	VEILTKQEGQR
KbNAP1B	115	K	N6-acetyllysine	0.646	VEILTKQEGQR
KbNAP1B	159	N	N-linked_glycosylation	0.913	LRYLTNITADP
KbNAP1B	228	S	Phosphoserine	0.537	DITIESVQKKR
KbNAP1B	282	T	Phosphothreonine	0.673	DDDEDTIDALM
KbNAP1B	282	T	O-linked_glycosylation	0.596	DDDEDTIDALM
KbNAP1B	358	K	SUMOylation	0.535	RKCGKKREKVK
ScNAP1	7	T	O-linked_glycosylation	0.543	SDPIRTKPKSS
ScNAP1	11	S	O-linked_glycosylation	0.602	RTKPKSSMQID
ScNAP1	12	S	O-linked_glycosylation	0.605	TKPKSSMQIDN
ScNAP1	20	T	Phosphothreonine	0.795	IDNAPTPHNTP
ScNAP1	20	T	O-linked_glycosylation	0.788	IDNAPTPHNTP
ScNAP1	24	T	Phosphothreonine	0.755	PTPHNTPASVL
ScNAP1	24	T	O-linked_glycosylation	0.759	PTPHNTPASVL
ScNAP1	27	S	O-linked_glycosylation	0.596	HNTPASVLNPS
ScNAP1	50	K	Ubiquitination	0.756	QEQDDKIGTIN
ScNAP1	76	S	Phosphoserine	0.840	QDRLGSLVGQD
ScNAP1	82	S	Phosphoserine	0.773	LVGQDSGYVGG
ScNAP1	140	S	Phosphoserine	0.806	RSRIISGQEQP
ScNAP1	159	S	Phosphoserine	0.785	QEIVESLNETE
ScNAP1	161	N	N-linked_glycosylation	0.890	IVESLNETELL
ScNAP1	175	N	N-linked_glycosylation	0.888	EEKAQNDSEEE
ScNAP1	177	S	Phosphoserine	0.847	KAQNDSEEEQV
ScNAP1	237	S	Phosphoserine	0.518	LFRFDSSANPF

Protein ID	Position	Residue	Predicted PTM	Score	Sequence Motif
ScNAP1	282	N	N-linked_glycosylation	0.909	KDNAH**N**VTVDL
ScNAP1	294	N	N-linked_glycosylation	0.900	MRKQR**N**KTTKQ
ScNAP1	320	K	Ubiquitination	0.571	FFDPP**K**IQNED
ScNAP1	342	Y	Phosphotyrosine	0.514	RLALD**Y**SIGEQ
ScNAP1	343	S	Phosphoserine	0.748	LALDY**S**IGEQL
ScNAP1	397	S	Phosphoserine	0.842	DDDGE**S**AEEQD
ScNAP1	397	S	O-linked_glycosylation	0.783	DDDGE**S**AEEQD

Table S5. Oligonucleotide primers used in this study.

Applications	Target	Primer name	Sequence (5′ to 3′)
Cloning of NAP1	5′ region of NAP1	CSL	CCGTAGCCATTTGGCTCAAG
	3′-region of NAP1	Oligo(dT)-adaptor	GGTCTGAGGTCGTCTATGCTTTT TTTTTTTTTTTT
	KbNAP1A	Kb-NAP1A-F1	GTGGCATGGTCGAACCCTGC
	KbNAP1A	Kb-NAP1A-R1	GCCTTCCAATGGCGAAGCGC
	KbNAP1B	NAP1B-ENPYF-10-F	GTTTTCCCAGTCACGACGARAA YCCNTAYTT
	KbNAP1B	NAP1B-WYTGEA-151-R	CAGGAAACAGCTATGACGCYTC NCCNGTRTACCA
Recombinant protein expression	CcH2AX.2 (93-199 aa; antibody antigen)	pQE30-H2A(93-199)-F	ATATGGATCCATCGGGCGCTTC ACGAAGCAGCTG
	CcH2AX.2 (93-199 aa; antibody antigen)	pQE30-H2A(93-199)-R	ATATAAGCTTTCAGAACTCTTGA GACATCTCAAC

The highly conserved nucleosome assembly protein 1 (NAP1) performs diverse roles across eukaryotes, yet its precise biochemical function, particularly its proposed role as a histone chaperone, remains unclear. Dinoflagellates, which lack architectural nucleosomes and express histones at unusually low levels, nonetheless retain abundant NAP1 transcripts encoding two distinct homologs. Confocal immunolocalization showed strong nuclear signals for both homologs between chromosomes and at the nucleolus, with higher nuclear-to-cytoplasmic ratios in G_2 than G_1; enrichment at chromosome territory margins and nuclear cortices suggested association with telomeric nucleosomes anchored to the nuclear envelope. Biochemically, KbNAP1Bp retained the canonical H2A:H2B-binding interface, co-immunoprecipitated with H2A, and exhibited size-selective, volume-depletion-sensitive DNA binding, while KbNAP1Ap lacked H2A binding and instead bound DNA and dHLPs non-selectively, a functional divergence supported by structural modelling. Although both homologs co-localized in transcriptionally active peripheral chromosome loops and nucleoli, their physical association with dHLPs, the dominant chromosomal proteins of the Hup-linker histone superfamily, was weak. NAP1 abundance peaked during S-G_2, and antisense knockdown caused a corresponding cell cycle delay. These findings support a model in which dinoflagellate NAP1 retains a residual, isoform-specific

histone chaperone function alongside a broader role in maintaining chromosome territory organization.
